# Electron Trap Depths in Cubic Lutetium Oxide Doped with Pr and Ti, Zr or Hf—From Ab Initio Multiconfigurational Calculations

**DOI:** 10.1021/acs.jpca.2c07979

**Published:** 2023-05-17

**Authors:** Andrii Shyichuk, Marek Krośnicki

**Affiliations:** † Faculty of Chemistry, University of Wrocław, ul. F. Joliot-Curie 14, 50-383 Wrocław, Poland; ‡ Institute of Theoretical Physics and Astrophysics, Faculty of Mathematics, Physics and Informatics, University of Gdańsk, ul. Wita Stwosza 57, 80-308 Gdańsk, Poland

## Abstract

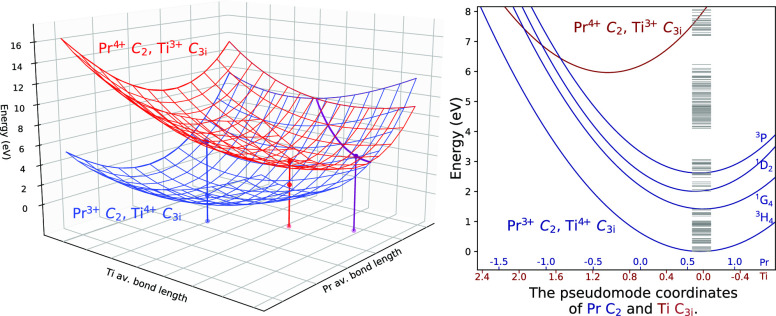

We propose a universal approach to model intervalence charge transfer (IVCT) and metal-to-metal charge transfer (MMCT) transitions between ions in solids. The approach relies on already well-known and reliable ab initio RASSCF/CASPT2/RASSI-SO calculations for a series of emission center coordination geometries (restricted active space self-consistent field, complete active space second-order perturbation theory, and restricted active space state interaction with spin-orbit coupling). Embedding with ab initio model potentials (AIMPs) is used to represent the crystal lattice. We propose a way to construct the geometries via interpolation of the coordinates obtained using solid-state density functional theory (DFT) calculations for the structures where the activator metal is at specific oxidation (charge) states of interest. The approach thus takes the best of two worlds: the precision of the embedded cluster calculations (including localized excited states) and the geometries from DFT, where the effects of ionic radii mismatch (and eventual nearby defects) can be modeled explicitly. The method is applied to the Pr activator and Ti, Zr, Hf codopants in cubic Lu_2_O_3_, in which the said ions are used to obtain energy storage and thermoluminescence properties. Electron trap charging and discharging mechanisms (not involving a conduction band) are discussed in the context of the IVCT and MMCT role in them. Trap depths and trap quenching pathways are analyzed.

## Introduction

Long-term energy storage and thermoluminescence in phosphors based on cubic Lu_2_O_3_:Pr can be achieved via the introduction of d metal codopants such as Hf,^1−4^ Nb,^5^ and Ti.^5,6^ Under ultraviolet (UV) light irradiation, the Pr^3+^ dopant is excited to its 4f^2^ and 4f^1^5d^1^ excited states. Some of those states overlap with the conduction band of the matrix material (*Ia*3̅ Lu_2_O_3_).^7,8^ A Pr^3+^ → Pr^4+^ photoionization is thus possible upon Pr^3+^ f–d or f–f excitation. The formation of Pr^4+^ was suggested by Wiatrowska and Zych^3^ from the respective decrease of Pr^3+^ f–d UV absorption in the X-ray-irradiated Lu_2_O_3_:Pr,Hf (i.e., upon high-energy excitation, some part of Pr^3+^ were converted to Pr^4+^). The d orbitals of the codopant (Hf) were assumed to trap the resulting electron,^2^ although this was not the only assumed electron trapping mechanism. The same work showed that even without the codopant, Lu_2_O_3_:Pr can exhibit thermoluminescence—albeit very weak. X-ray absorption near-edge structure (XANES) X-ray absorption experiments^9^ show the presence of both Pr^3+^ and Pr^4+^ in Lu_2_O_3_:Pr,Hf and Lu_2_O_3_:Pr, indicating that Pr^4+^ is stable in these materials.

The following is a commonly accepted thermoluminescence/energy storage mechanism in Lu_2_O_3_ phosphors. Before the irradiation (in its state without the trapped electron), the codopant is assumed to be in its higher oxidation state (e.g., Hf^4+^). After the excitation and subsequent Ln^3+^ → Ln^4+^ ionization (Ln = Pr, Tb), the electron released at the former 3+ Ln site is assumed to travel via a conduction band until it reaches a trap site. The trap sites are characterized by a separated energy level below a conduction band and are typically attributed to the codopant. After the excitation, the codopant is reduced (to Hf^3+^, for example) by the electron received from the conduction band. The reduced codopant remains in that oxidation state until it receives a portion of energy high enough to promote the electron back to the conduction band.

Depending on the codopant, the electron traps in Lu_2_O_3_:Pr can have substantially different depths.^5,6^ For the shallow traps corresponding to the thermoluminescence glow curve peaks below 200 °C,^2^ visible afterglow is observed after UV irradiation of Lu_2_O_3_:Pr,Hf, for example. With the increase in the depth of the traps, permanent energy storage can be achieved. In the latter case, once the material is irradiated, it can forever store a portion of the excitation energy. Similarly, Lu_2_O_3_:Tb can be codoped with transition metal cations. Given the appropriate synthesis conditions, a Lu_2_O_3_:Tb phosphor with thermoluminescence properties can be made. From the experimental data collected for Lu_2_O_3_:Tb,M (M being Ti,^10,11^ Zr,^11^ Hf,^10−14^ V,^15^ Nb,^10^ and Ta^16,17^) and Lu_2_O_3_:Pr,M,^1−6^ it occurs that the codopant is more crucial in defining the electron trap character. For instance, the most intense thermoluminescence glow peak is located at roughly the same temperatures^5^ for either Lu_2_O_3_:Tb,M or Lu_2_O_3_:Pr,M: at 370 °C for M = Ti, 230–250 °C for M = Hf. A noteworthy case was shown in the work by Sójka et al.,^11^ where Zr and Hf codopants resulted in practically identical thermoluminescence in Lu_2_O_3_:Tb,M. Between Pr and Tb, the Pr dopant was selected as the subject of this paper as it is significantly simpler to treat computationally due to the small number of valence electrons—namely, two for Pr^3+^ and one for Pr^4+^.

Despite the vast experimental data, a particular mechanism of energy storage and trap depopulation in Lu_2_O_3_:Ln,M is not completely clarified. Albeit likely, it is not directly confirmed that all of the mentioned codopants act as electron traps (i.e., if the dopant cations get locally reduced to form metastable trap states). Moreover, recent studies for Lu_2_O_3_:Tb,M phosphors indicate that some of the depopulation processes likely involve the trapped electron excitation to the conduction band,^18^ while the others do not—a direct transfer of the electron to the Tb^4+^/Tb^3+^ recombination center occurs.^19^ In this research, we have tackled the charging–discharging (electron trapping and release) mechanism using advanced post-Hartree–Fock correlated electron calculations (multiconfigurational and coupled cluster). Several practical issues were resolved, giving a rather straightforward and universal approach to estimating the trap depth for a metal-to-metal charge transfer (MMCT) mechanism of trap population and depopulation. In other words, we have considered a direct electron exchange between the dopant and the codopant without a conduction band involved. Ti, Zr, and Hf codopants were selected: they feature only two oxidation states each (+3 and +4) and correspond to a broad range of traps (Ti: deep; Zr, Hf: moderate and shallow). Additionally, as the [Ln^3+^, M^4+^] and [Ln^4+^, M^3+^] ionic pairs are symmetric in terms of local uncompensated charge (+1 at one of the dopants, zero at the other, both in Lu^3+^ sites), no additional energy shifts (due to electron–hole attraction) are needed.^20^ Identical traps corresponding to Lu_2_O_3_:Tb codoping with either Hf or Zr^11^ were another reason to compare Zr and Hf electron trapping properties.

To model the thermoluminescence emission mechanism in Lu_2_O_3_:Pr,M, the appropriate emission levels of Pr^3+^ had to be selected. In cubic lanthanide sesquioxides, Pr^3+^ does not exhibit green emission from ^3^P/^1^I manifolds (the matter revised by Srivastava et al.^8^ and references therein). Instead, red emission from ^1^D_2_ is observed. This phenomenon is explained by cross-relaxation by Pedroso et al.^9^ Srivastava et al. explain it by the overlap of the ^3^P and ^1^I manifolds with the conduction band.^8^ Both mechanisms result in efficient quenching of the ^3^P/^1^I manifold and the consequent emission from ^1^D_2_. In the work by Kulesza et al.,^4^ a thermoluminescence emission spectrum of Lu_2_O_3_:Pr,Hf ceramics is shown in the 450–800 nm range, indicating only red emission peaks at 600–700 nm and no green emission. The Pr^3+^ red thermoluminescence emission in the 600–700 nm range was attributed to transitions from the ^1^D_2_ manifold by Wiatrowska et al.^2^ Kulesza et al. show^5^ that both optically stimulated Pr^3+^ emission (upon optical emptying of the previously charged traps) and regular UV–vis Pr^3+^ emission spectra are very similar to those of the thermoluminescence Pr^3+^ emission. In other words, no matter the population mechanism, the Pr^3+^ emission in Lu_2_O_3_ is always red.

Noteworthy, Toncelli et al.^21^ attribute this emission to transitions from the ^3^P manifold, which is not surprising. Using the levels from the said paper and all of the permutations between them, it is possible to find transitions from both ^3^P and ^1^D_2_ (to the ^3^H_3–4_ levels) that match the 600–700 nm peaks, while the selection rules predict higher intensities for the spin-allowed triplet–triplet transitions from ^3^P. A similar conclusion can be made from energy levels obtained in this work—from energy differences alone, both ^3^P and ^1^D_2_ levels can be the initial ones of the emission in question. Quenching processes must therefore define the observed emission. In summary, we are inclined to assume that ^1^D_2_ is the initial level of interest. However, we have also analyzed the trap depths for the ^3^P/^1^I population.

## Methods

The model of energy storage applied in this work is based on the following ideas. In its uncharged (inactivated, unperturbed) state, the thermoluminescence (storage) Lu_2_O_3_:Pr,M (M = Ti, Zr, Hf) phosphor contains (from a very localized, microscale, atom-based point of view) a Pr^3+^ ion in an Lu^3+^ site and an M^4+^ ion in an Lu^3+^ site. Upon excitation, Pr^3+^ gets ionized to Pr^4+^, and the produced electron is transferred to the M^4+^ ion—which is reduced to M^3+^. Provided there is a barrier for the reverse process, the system will stay in this metastable charged (activated, Pr^4+^ + M^3+^) state until the energy sufficient for the electron to overcome the barrier is delivered to the system. In other words, the electron is trapped at the M^3+^ site. The model used does not include a conduction band and hence corresponds to a metal-to-metal charge transfer (MMCT) mechanism of the electron trapping/detrapping processes.

### Dopant Ion Clusters and Pairs

The part of the Lu_2_O_3_:Pr,M (M = Ti, Zr, Hf) materials that was modeled explicitly was the dopant cation and the oxygens of its immediate surroundings. The rest of the lattice was represented using embedding potentials. Such an approach assumes that the corresponding dopant clusters in the real material are separated by a certain distance—such that any deformations in the geometry of one cluster do not affect the other cluster. As the thermoluminescence materials of interest are characterized by a rather low dopant content,^5^ such an assumption is entirely acceptable. Consequently, independent calculations for each cluster geometry were performed. We have also considered the case of the two dopants occupying nearest neighbor sites. That requires a large embedded cluster containing both dopant ions. Such a case is methodologically quite different from what is presented below and is much more complex and extended. It was thus not included in the current paper.

To estimate the amounts of energy required to populate the trap (convert a [Pr^3+^, M^4+^] pair into a [Pr^4+^, M^3+^] pair) and to uncharge the trap (convert the [Pr^4+^, M^3+^] pair into the [Pr^3+^, M^4+^] pair), the total electronic energies of the pairs need to be calculated. We follow the idea of Barandiarán and Seijo, discussed in detail in the respective papers.^22,23^ This idea was recently successfully applied to the whole lanthanide series^24^ and electron trapping in particular.^25^ All of the mentioned papers, however, feature highly symmetric CaF_2_-type cubic structures, and it is problematic to apply their methodology to a less symmetric Lu_2_O_3_ directly.

Activation of the [Pr^3+^, M^4+^] pair via electron transfer into [Pr^4+^, M^3+^] is associated with changes in both Pr and M oxidation states followed by relaxation of geometries of the activated pair. We model this process by calculating diabatic electronic energies of the cluster pairs for a set of geometries representing a transformation between the activated [Pr^4+^, M^3+^] pair and deactivated [Pr^3+^, M^4+^] pair, as described in the following sections. The total diabatic electronic energy of the charged/activated [Pr^4+^, M^3+^] pair is represented by the sum of the adiabatic electronic energies of PrO_6_^8–^ and MO_6_^9–^ separated clusters. Please note that the cluster–cluster coupling energy is not calculated: the energy (diabatic) of a cluster pair is a sum of the separate noninteracting cluster adiabatic energies at a particular reaction coordinate. This means that diabatic electronic energy surfaces will not provide quantitative energy barriers in the case of avoided crossings of the cluster pair electronic surfaces. The surfaces, however, let us follow the chemical character of the cluster pair electronic states along the reaction coordinate and estimate energy barriers from diabatic crossings.

### Potential Energy Surfaces

In the assumed mechanism of ion–ion interactions (MMCT in particular), two ions change their oxidation states upon electron transfer. That, in turn, must result in non-negligible changes in bond length between the ions and the surrounding oxygens. When a geometry of a single cluster is changed in correspondence with a vibrational mode (along a linear reaction coordinate), the electronic energy of the cluster changes. This change can be plotted as a function of the reaction coordinate, which forms a potential energy curve. The process of electron transfer involves two separate clusters and thus depends on two independent reaction coordinates. In a [PrO_6_^8–^, MO_6_^9–^↔ PrO_6_^9–^, MO_6_^8–^] electron transfer process, both the Pr–O and M–O bond lengths change. The activated [Pr^4+^, M^3+^] pair is composed of PrO_6_^8–^ and MO_6_^9–^ clusters, while the inactivated [Pr^3+^, M^4+^] pair is composed of PrO_6_^9–^ and MO_6_^8–^ clusters. One reaction coordinate describes the transformation between the PrO_6_^8–^ (Pr^4+^) and PrO_6_^9–^ (Pr^3+^) clusters, while the other coordinate describes a transformation between the MO_6_^9–^ (M^3+^) and MO_6_^8–^ (M^4+^) clusters. The energy of a pair of clusters as a function of the respective reaction coordinates consequently forms a potential energy surface. The same coordinates are used to construct diabatic energy surfaces of both the [Pr^4+^, M^3+^] and the [Pr^3+^, M^4+^] pairs. This is possible because the clusters of the ion pairs are considered as not-interacting in this model.

Let us denote the diabatic electronic energies of the ion pair as *z* = *E*(*x*, *y*), where the Pr^4+^/Pr^3+^ reaction coordinate is used as *x*, and the M^3+^/M^4+^ reaction coordinate is used as *y*. In particular, the diabatic potential energy surface of the activated/charged [Pr^4+^, M^3+^] pair is defined as . The surface of the inactivated/uncharged [Pr^3+^, M^4+^] pair is . The surfaces intersect along a line that (usually) has a minimum (at the proximity of the studied system minima). The difference between the minimum of the *E*_*I*_ surface of the charged system [Pr^4+^, M^3+^] and the minimum of the *E*_*I*_/*E*_*II*_ surface intersection is taken as the trap depth in this model.

### Cluster Geometries and Pseudomodes

In either thermal detrapping or a more general MMCT process, the 3+ and 4+ equilibrium geometries of a particular ion must transform into each other—i.e., atoms of the ion’s surroundings have to move in response to the change in the charge state of the said ion. Thus, a coordinate path transforming the XO_6_^9–^ equilibrium geometry into the XO_6_^8–^ equilibrium geometry (X = Pr, Ti, Zr, Hf) must exist. We have proposed making a linear interpolation between the X^3+^/X^4+^ equilibrium geometries to exemplify this coordinate path. As stated before, a *z* = *E*(*x*, *y*) potential energy surface is a function of two independent reaction coordinates *x* and *y*: the former corresponding to the Pr^3+^/Pr^4+^ coordinate path, and the latter corresponding to the M^3+^/M^4+^ coordinate path (M = Ti, Zr, Hf).

To construct the respective coordinate path, the initial and final geometries of the clusters were obtained using density functional theory (DFT, the next subsection). Having those, it is possible to construct a transformation numerically analogous to a vibrational mode, such that it transforms the cluster geometries (of the same atom at different oxidation states) into each other. We have called such a transformation a pseudomode. It is assumed to represent a thermally induced transition and, thus, might be a part of a more extensive (nonlocal, phonon) vibrational mode. The utilized pseudomodes have produced smooth (parabola-shaped) potential energy curves at the RASSCF level of theory.

Given a Cartesian coordinate matrix **G**_1_ (representing, for example, equilibrium PrO_6_^8–^ geometry, Pr^4+^) and a Cartesian coordinate matrix **G**_2_ (representing, for example, equilibrium PrO_6_^9–^ geometry, Pr^3+^), the Cartesian displacement matrix of the pseudomode is given by **D** = **G**_2_ – **G**_1_. The interpolated geometries **G**_*u*_ for a potential energy curve that uses the pseudomode are created using a scalar parameter *u* (a pseudomode coordinate): **G**_*u*_ = *u***D** + **G**_1_. Setting *u* = 1 gives **G**_2_, *u* = 0 corresponds to **G**_1_, and 1 > *u* > 0 corresponds to the intermediate (interpolated) geometries, while the other values of *u* give the extrapolated geometries. The same principle was used for M^3+^ and M^4+^ geometries.

Having a pair of Pr and M ions and the 3+ and 4+ equilibrium geometries for each of them (, ; , , obtained from the DFT calculations), the **D**_Pr_ and **D**_M_ transformations are
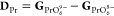
1
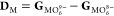
2

Those displacement matrices define geometries ,  for PrO_6_^8–^, PrO_6_^9–^ and MO_6_^8–^, MO_6_^9–^ clusters, respectively. The diabatic potential energy surfaces of activated/deactivated systems can now be expressed using parameters *u*_Pr_ and *u*_M_ of the respective pseudomodes instead of the arbitrary *x* and *y*:
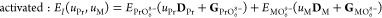
3
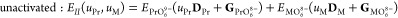
4

The energies in the equations above were obtained for a series of interpolated geometries **G**_Pr_ and **G**_M_, using ab initio calculations described in the following section. Each of the obtained potential energy curves (of the individual clusters) was fitted with a cubic polynomial to get the respective analytical representation of the curves. The intersection of the *E*_*I*_ and *E*_*II*_ surfaces (defined in eqs 3 and 4, respectively) was found analytically using the cubic roots formula. The details are provided in the Supporting Information (in the Python script that does the analysis and plotting). The intersection of the two surfaces is a nonplanar three-dimensional curve. The minimum of the intersection curve was used to estimate the trap depths and other energetic parameters.

There are several factors motivating the transformation of cluster geometries along pseudomodes. The approach involving intersecting diabatic surfaces has been used before in intervalence^22,23^ and metal-to-metal^20,24,25^ charge transfer studies. However, those studies featured highly symmetric CaF_2_-type lattices, where activator site geometry optimization was not required: vibrational deformations were addressed via simple proportional scaling of the whole cube surrounding the metal ion, corresponding to a full-symmetric (breathing) vibrational mode of the activator site. The structure of cubic lutetium oxide is more complex:^26^ there are two six-coordinated sites of C_3*i*_ and C_2_ local symmetries, both of which look like distorted cubes with two unoccupied vertex sites.

A metal-to-metal charge transfer process means that every involved ion changes its oxidation state. For the dopant ions, there are two charge states (before and after) and two site symmetries to be considered. For each of those, the equilibrium geometries of the coordination polyhedron can be different in terms of both bond length and bond angles. Unlike CaF_2_-type lattices, the individual before and after geometries do not necessarily transform into each other via scaling of the bond lengths.

For every ion in question and for every site symmetry of the c-Lu_2_O_3_ lattice, normal vibrational modes were obtained and analyzed for the embedded clusters. It turned out that for the same ion and site symmetry, none of the 4+ ion cluster modes can transform (morph) the 4+ ion equilibrium geometry into the 3+ ion equilibrium geometry. The same is true for the 3+ ion cluster modes and 4+ ion cluster geometry. In other words, for the same ion and site symmetry, the normal mode vibrational coordinates of the 3+ ion cluster do not intersect with the normal mode vibrational coordinates of the 4+ ion cluster at any point. The geometries produced by the respective deformations do get close to each other in some cases but are never identical. The pseudomode coordinate path solves this issue, making two arbitrary geometries transform into each other by construction.

### DFT Calculations

The geometries of the clusters (namely, PrO_6_^9–^, PrO_6_^8–^, MO_6_^8–^ and MO_6_^9–^) were obtained using plane-wave density functional theory calculation (DFT) on a doped unit cell of Lu_2_O_3_, doing a full cell relaxation (for both 3+ and 4+ dopants). The input geometry from the paper by Zeler et al.^26^ was used. GBRV^27^ ultrasoft pseudopotentials for Ti, Zr, and Hf and Topasakal and Wentzcovitch^28^ projector augmented wave (PAW) potentials for Pr were used. PBEsol^29^ exchange-correlation functional was utilized. The code was the PW module of Quantum Espresso.^30,31^ The detailed description of the code parameters and functional selection is given in the Supporting Information.

For the Hf-doped systems, two independent sets of calculations were performed, with the the only difference between the two being the Hf pseudopotential. Hafnium was regarded as a problematic element by Garrity et al.:^27^ namely, a universal Hf^0^ ultrasoft pseudopotential did not perform well as Hf^4+^ in different oxides and, thus, a dedicated Hf^4+^ ultrasoft pseudopotential has been introduced. The pseudopotentials differ, among others, by their valence shells: both have occupied 5s and 5p orbitals and empty 6s and 6p orbitals; the Hf^0^ pseudopotential was made with two electrons in 5d orbitals, while the Hf^4+^ pseudopotential has empty 5d orbitals and additional 5f empty orbitals. In our case, an Hf^3+^ ion in an oxide should be more similar to an Hf^4+^ ion rather than a neutral Hf. However, just to be safer, we made two sets of calculations using each of the pseudopotentials. As a result, two slightly different cell geometries were obtained, and the two sets of data are present.

### Ab Initio Calculations

The following post-Hartree–Fock correlated electron calculations were performed with the OpenMOLCAS^32,33^ software package. The clusters were placed in the embedding that represented the unperturbed lattice of c-Lu_2_O_3_: a 12 Å layer of ab initio model potentials (AIMPs)^34−36^ surrounded the MO_6_ clusters, followed by a layer of point charges with the external radius of about 77 Å. The point charges were optimized to minimize the values of electric multipoles (of orders 2, 3 and 4) at the cluster atoms using Lattgen code.^37^ This kind of embedding was used to optimize the AIMPs in a self-consistent embedding ion procedure.^38^ The ready-to-use embedding files are given as Supporting Information. For each specific geometry of each cluster, the calculations began with relativistic Douglas–Kroll–Hess^39^ integrals (order of Hamiltonian 2, order of properties 2, SEWARD code^40,41^). Self-consistent field calculations followed (SCF code,^42−46^ Hartree–Fock, without the active electrons, i.e., on clusters containing Pr^5+^, Ti^4+^, Zr^4+^, Hf^4+^: PrO_6_^7–^ and MO_6_^8–^). The basis set was ANO-RCC triple-ζ.^47−49^

The next step depended on the metal cation of the cluster. The potential energy curves for the ground state of the M dopants were calculated using the CCSD(T)^50^ calculation on top of a single-root ground state restricted active space (RASSCF^51−53^) wave function (using single-orbital active space, effectively an SCF calculation). For the M^3+^ systems, full T2 and T1 spin adaptation (according to Raghavachari et al.^54^) was utilized. In the noniterative triples procedure, the denominators were the diagonal Fock matrix elements (closed-shell M^4+^) or the orbital energies (open-shell M^3+^).

For Pr clusters, state-average RASSCF calculations followed the Hartree–Fock step. The active orbitals (RAS2 orbitals in MOLCAS notation) were molecular orbitals with a predominant Pr 4f, 6s and 5d character. In the case of Pr^3+^/PrO_6_^9–^, two electrons populated the active space. In the case of Pr^4+^/PrO_6_^8–^, one electron populated the active space. All possible roots were included (i.e., the number of roots [states] for the state-average calculation was equal to the number of configuration state functions). For Pr^3+^, singlet and triplet states were considered. For Pr^4+^, doublet states were calculated. Next, restricted active space second-order perturbation theory (RASPT2^55−57^) calculations followed. For Pr^3+^, only the most significant roots (all of the 4f^2^ and 4f^1^5d^1^ states) were included due to calculation stability issues. What is noteworthy is that the zeroth-order wave function reference weights in the RASPT2 calculations were typically 0.64–0.67. The frozen core orbitals were O 1s; Hf, Pr 1–4s, 2–4p, 3d, 4d; Ti 1–2s, 2p; Zr 1–3s, 2–3p, 3d—as recommended by the basis set authors.^47−49^ The IPEA shift was set to zero. The imaginary shift^58^ of 0.3 was used for Pr (required to get smooth Pr^3+^ potential energy curves at the RASPT2 level of theory). Finally, RASSI-SO^59,60^ calculations were used to include the effects of spin–orbit coupling and to couple the states of different irreducible representations and spin multiplicities: for the C_2_ sites, representations A and B were coupled, while for the C_*i*_ site, representations A_g_ and A_u_ were mixed.

### Pr^3+^ Electronic State Selection

The potential energy surfaces were represented by the following: *E*_*I*_(*u*_Pr_, *u*_*M*_) (eq 3) is calculated as the energy of PrO_6_^9–^ selected RASSI-SO state plus MO_6_^8–^ CCSDT energy; *E*_*II*_(*u*_Pr_, *u*_*M*_) (eq 4) is calculated as the energy of PrO_6_^8–^ RASSI-SO ground state plus MO_6_^9–^ CCSDT energy. The input cluster geometries corresponded to the respective *u*_Pr_ and *u*_*M*_ pseudomode coordinates.

The selection of a particular Pr^3+^ electronic state (calculated on the RASSI-SO level theory) for the surface intersection analysis depended on the analyzed energetic property. For example, a detrapping followed by the emission, or a trap quenching corresponds to different Pr^3+^ states after the [Pr^4+^, M^3+^] → [Pr^3+^, M^4+^]. For one of the quenching cases, the Pr^3+^ ground state was picked. For other processes, the most relevant excited states were selected. One of them was the top of the ^1^*G* manifold, as deduced from the energy gaps between states. These two cases corresponded to the final Pr^3+^ states that resulted in no Pr^3+^ red emission (i.e., the nonradiative loss of the trapped electron energy). To get the trap depth corresponding to the experimentally observed red emission in the 600–700 nm range (described by, e.g., Wiatrowska and Zych^2^), the respective excited state had to be selected. As shown in the Introduction, such a selection is not straightforward. We selected the lowest level of the ^1^D_2_ manifold—it was also the first level above the ^1^G manifold. That was the lowest Pr^3+^ level from which the red emission is still possible. The level is referred to below as ^1^D_2_. For comparison, we have also selected a higher level from which green emission should be possible. For the C_2_ site, we picked a level with relatively high spontaneous emission coefficients (calculated with RASSI-SO). By the singlet–triplet character, it is likely a ^3^P level. For the C_3*i*_ site, however, the coefficients were exactly zero due to inversion symmetry. We have consequently picked the first level above the ^1^D_2_ manifold, which is mostly singlet and is, thus, likely a ^1^I level.

## Results and Discussion

### Calculated Energy Levels

**Figure 1:**
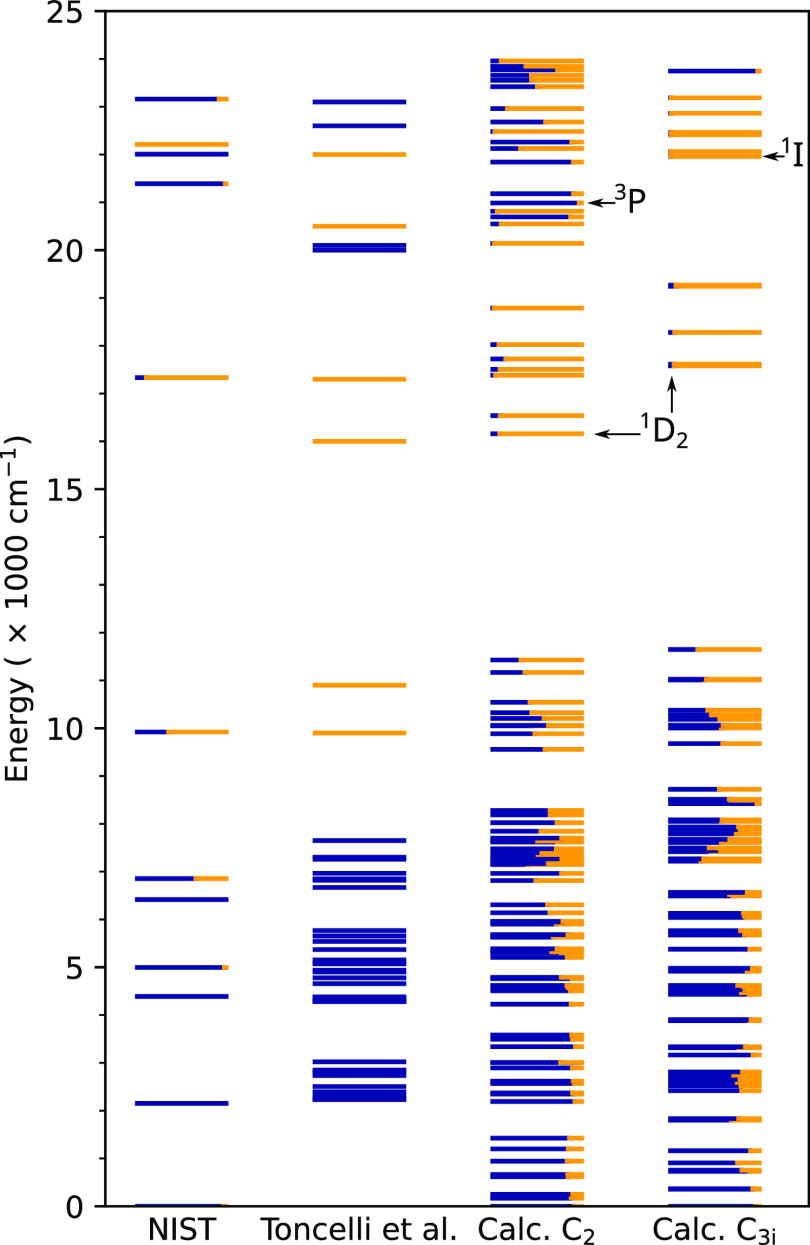
Pr^3+^ free-ion levels from NIST,^61^ Lu_2_O_3_:Pr^3+^ experimental levels from optical absorption measurements by Toncelli et al.,^21^ and the calculated RASSI-SO levels from this work. The blue and orange parts of the level bars are proportional to their triplet and singlet characters, respectively. The term symbols indicate which particular levels are referred to in the text.

The calculated energy levels (that included spin–orbit coupling, the RASSI-SO states) for the two Pr^3+^ sites, the NIST Pr^3+^ free-ion energy levels^61^ and the experimental Lu_2_O_3_:Pr^3+^ energy levels from ref (21) are shown in Figure 1. The percentage of singlet and triplet contributions to the SO states are visualized using colors (Figure 1). Term symbols and arrows indicate the states selected for the trap depths analysis, which were the final Pr^3+^ states of a [Pr^4+^, M^3+^] → [Pr^3+^, M^4+^] transition. The calculated energy levels correspond well to the experimental ones (Toncelli et al.^21^), while the triplet/singlet character is a good match to that of the NIST levels.^61^ The calculated energy levels are also in accord with the ones reported by Pascual et al.,^62^ where the gap between the ^1^D and ^3^P/^1^I manifolds is noticeably larger for Pr^3+^ in the C_3*i*_ site than that of the C_2_ Pr^3+^ (Figure 1).

### Energy Barriers: Error Estimation

It is worth mentioning that the calculated trap depths (and other energetic parameters) should correspond to the actual thermally driven processes in the real materials—from the purely physical-chemical microscopic perspective. We do not know for a fact that the pseudomode coordinate is the optimal path to the barrier. It is, however, a necessary approximation and very low-cost at that (compared to a hypothetical search for the lowest-energy path).

Certain unavoidable errors always emerge from the method’s intrinsic limitations. In our particular case, the energy surfaces are diabatic (i.e., there is no cluster–cluster interaction explicitly modeled). Basically, all the crossings between diabatic energy surfaces of different electronic states of a cluster–cluster pair are allowed. We omit a description of the avoided crossings and conical intersections, which would be present in the adiabatic representation of the cluster–cluster pair. Therefore, our results are upper boundary estimates of the energy barrier heights.

On the other hand, even if the calculated trap depths were error-free, their values do not necessarily translate to the macroscopic thermoluminescence kinetics equations.^63^ In the latter, the trap depth has a certain fit parameter flavor to it: it depends on the selected kinetics order, to say the least. Accordingly, trap depths from these calculations should be handled with care in the context of their comparison to the experimental trap depths.

### Metal-to-Metal Charge Transfer and Localized Trapping

**Figure 2:**
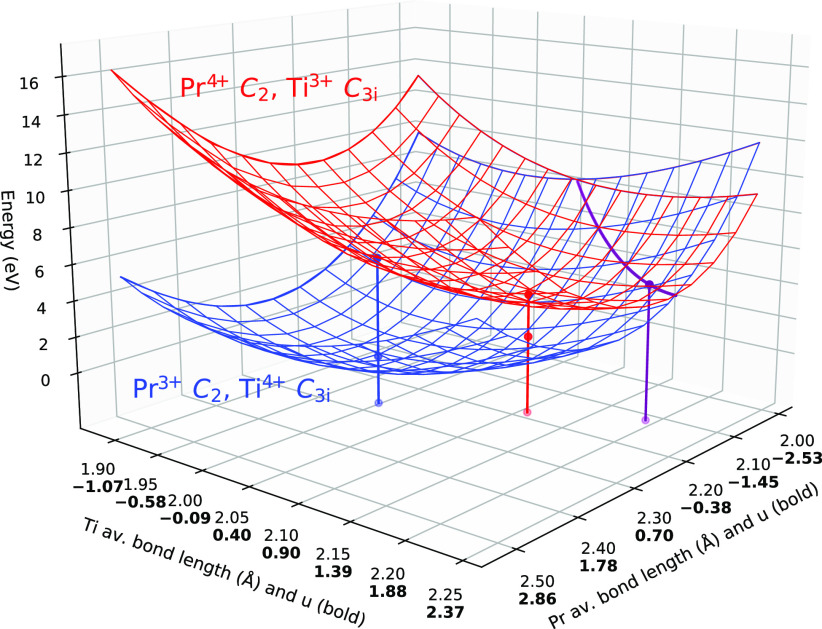
Example of the intersecting potential energy surfaces used to determine the trap depth and optical gaps.

Each pair of the intersecting surfaces in question has its own unique look and properties. However, they are all similar in principle and feature two paraboloid-like surfaces and an intersection line. For clarity, we show only one of them in Figure 2, where the features are the most evident. Any curious reader is invited to use the supplementary data files and the attached Python script to view the respective interactive 3D plots.

The calculated trap depths, optical activation energy gaps and other energetic properties for the dopant pairs and local symmetries in question are summarized in Table 1 below. Each trap depth (energy barrier) was obtained from the respective *E*_*I*_(*u*_Pr_, *u*_*M*_) and *E*_*II*_(*u*_Pr_, *u*_*M*_) (eqs 3 and 4) potential energy surface intersection minimum and the activated (filled trap) [Pr^4+^, M^3+^] pair (*E*_*I*_) minimum. This depth corresponds to a thermally (vibrationally) induced metal-to-metal charge transfer transition from the activated [Pr^4+^, M^3+^] pair minimum (*E*_*I*_ minimum, the trapped electron state) to the minimum of the intersection line between the [Pr^4+^, M^3+^] and [Pr^3+^, M^4+^] (*E*_*I*_(*u*_Pr_, *u*_*M*_) and *E*_*I*_*_I_*(*u*_Pr_, *u*_*M*_)) potential energy surfaces. In Figure 2, that would be a transition from the red point to the purple point along the red surface. Note that in the latter case, the [Pr^3+^, M^4+^] pair corresponds to the state after the electron detrapping and recombination, where the Pr^3+^ (the recombination center) is either in the bottom of its ^1^D_2_ manifold or in one of the higher ^1^I (the C_3i_ site) or ^3^P (the C_2_ site) levels.

**Table 1:** Energetic Properties of Trapping and Detrapping Processes from the CCSDT/RASSI PES and Their Intersections^a^

		TD (eV)		QB (eV)		OTPG		QG/UOE
Ions, site symm.		^1^D_2_	^3^P/^1^I	^1^G_4_	^3^H_4_	(eV)	(nm)	(eV)
Ti C_3*i*_	Pr C_3*i*_	0.55	0.87	0.26	0.00	4.10	302.3	–0.07
Ti C_2_	Pr C_3*i*_	3.46	4.26	2.51	1.08	0.78	1598.4	–1.000
Ti C_3*i*_	Pr C_2_	0.91	0.43	1.16	2.85	7.68	161.5	–4.27
Ti C_2_	Pr C_2_	0.03	0.22	0.00	0.41	4.35	284.9	–1.50
Zr C_3*i*_	Pr C_3*i*_	0.02	0.12	0.01	0.46	5.34	232.0	–1.75
Zr C_2_	Pr C_3*i*_	3.01	3.89	2.01	0.66	0.92	1352.2	–0.51
Zr C_3*i*_	Pr C_2_	3.14	2.00	3.39	6.27	8.92	139.0	–5.94
Zr C_2_	Pr C_2_	0.02	0.03	0.10	0.91	4.49	276.0	2.20
Hf^b^ C_3*i*_	Pr C_3*i*_	0.01	0.02	0.09	0.78	5.84	212.4	–2.30
Hf^b^ C_2_	Pr C_3*i*_	2.47	3.11	1.55	0.38	1.23	1011.2	–0.09
Hf^b^ C_3*i*_	Pr C_2_	3.99	2.66	4.20	7.50	9.41	131.7	–6.49
Hf^b^ C_2_	Pr C_2_	0.13	0.00	0.27	1.26	4.80	258.2	–2.70
Hf^c^ C_3*i*_	Pr C_3*i*_	0.05	0.00	0.19	1.04	6.47	191.5	–2.68
Hf^c^ C_2_	Pr C_3*i*_	2.01	2.73	1.22	0.24	1.62	766.5	0.24
Hf^c^ C_3*i*_	Pr C_2_	4.53	3.11	4.72	7.96	10.05	123.4	–6.87
Hf^c^ C_2_	Pr C_2_	0.264	0.037	0.45	1.73	5.19	238.7	–3.01

a TD: Trap depth, the barrier energy for a [Pr^4+^, M^3+^] → [Pr^3+^, M^4+^] process; the excited state of Pr^3+^ is given in the header. QB: Quench barrier, the barrier energy of a [Pr^4+^, M^3+^] → [^1^G_4_/^3^H_4_ Pr^3+^, M^4+^] process. OTPG: Optical trap population gap, the energy difference of a vertical (optical) [Pr^3+^, M^4+^] → [Pr^4+^, M^3+^] transition resulting in a filled trap. QG: Quenching gap, the energy of a vertical (optical) [Pr^4+^, M^3+^] → [Pr^3+^, M^4+^] transition resulting in the electron detrapping (see text). UOE: Unidirectional oxidation energy, the energy of a [Pr^3+^, M^4+^] → [Pr^4+^, M^3+^] transition resulting in the electron at M^3+^ and an oxidized Pr^4+^ (see text). The number of significant figures have been selected to show the differences between the values.

b Geometries obtained with Hf^4+^ pseudopotential.

c Geometries obtained with Hf^0^ pseudopotential.

Using the same principle, trap quench barriers (QBs) were obtained. The QB is the barrier of a process that would detrap the electron and result in no visible emission from Pr^3+^. In Figure 2, the QB would also be the energy of a transition from the red point to the purple point along the red surface—although this time the blue surface represents Pr^3+^ in one of its nonemitting levels. If the quench barrier is low, filling the trap would result in fast thermal relaxation to [Pr^3+^, M^4+^], and thus, the trap will not contribute to the energy storage and thermoluminescence. In other words, QBs are the barriers for thermally induced nonradiative transition from the [Pr^4+^, M^3+^] minimum (the trapped electron state) to the intersection between of the [Pr^4+^, M^3+^] and [Pr^3+^, M^4+^] surfaces. Here, [Pr^3+^, M^4+^] is the state after the detrapping and recombination, where Pr^3+^ can be in its ground state—i.e., the detrapped electron energy is vibrationally lost as the system goes down along the [Pr^3+^, M^4+^] surface (blue surface in Figure 2). Another kind of quench barrier featured Pr^3+^ in the upper level of its ^1^G_4_ excited manifold. There can be no visible Pr^3+^ emission from that level or any of the below levels, meaning that the stored energy is nonradiatively lost.

For the dopant pairs in question, an electron can also be transferred in a vertical (surface-to-surface) transition (straight lines in Figure 2), in which the geometries of the two pairs are the same (i.e., the pseudomode coordinates *u*_Pr_ and *u*_*M*_ must be the same for both surfaces in order for the vertical transition to happen). In time scales of those transitions, atom positions are considered static. The optical trap population gap (OTPG, the energy gap of an MMCT absorption that results in a filled trap) is the energy difference of a vertical transition from the [Pr^3+^, M^4+^] energy minimum (*E*_*II*_ minimum, Pr^3+^ in its ground state) to the [Pr^4+^, M^3+^] system at the same geometry coordinates. In other words, OTPGs are the energies of optical absorption-induced metal-to-metal charge transfer transitions that result in the filled traps upon optical excitation.

Quite a few of the OPTG values in Table 1 lie in the 250–320 nm range, which is used to charge the traps in Lu_2_O_3_-based thermoluminescence materials. It is therefore possible that MMCT processes contribute to optical absorption: in Lu_2_O_3_:Pr, only one 250–300 nm broad band is present in photoluminescence excitation spectra,^9^ while in Lu_2_O_3_:Pr,Ti there are two bands, at about 230–270 nm and about 300–350 nm.^6^

Another form of a vertical transition is (non)radiative relaxation, provided that the final state potential energy surface is below the initial state potential energy surface at the configurational coordinates of the initial state minimum. The [Pr^4+^, M^3+^] system might relax to [Pr^3+^, M^4+^]—we call the respective energy difference a quenching gap (QG). Negative values in the respective column of Table 1 indicate that the quenching process can happen in the same Pr–M pair as the trapping. The values of the QG are always negative for at least one state of Pr^3+^, and the table lists the lowest values. This result is very important: it shows that the electron trapped at M^3+^ will always decay via a [Pr^4+^, M^3+^] → [Pr^3+^, M^4+^] metal-to-metal charge transfer not involving a conduction band (the final state of Pr^3+^ depends on the M codopants and the site symmetries). For most [Pr^4+^, M^3+^] filled trap states, there is always a [Pr^3+^, M^4+^] state of a lower energy at the same reaction coordinate (at least within reasonably small deviations in bond length from the minima, of about ±10%). None of the listed electron traps is stable. On the contrary, in, e.g., refs (24 and 25), the trap states feature a barrier to the emitting part of the system and do not feature a vertical quenching pathway. The results presented here correspond well to the experimental fact of very low optimal concentrations of the dopants required for thermoluminescence to work in lutetium oxide,^5^ typically much lower than 1%. While the energetics of the trapping and quenching do not depend on the distance between the interacting species (for these particular ones), the probabilities must. Lower concentrations mean lower average distances and, thus, lower quenching chances.

Another relaxation process is a transition from a [Pr^3+^, M^4+^] pair to a [Pr^4+^, M^3+^] pair, with the energy difference called the unidirectional oxidation energy (UOE). Negative values indicate that the [Pr^4+^, M^3+^] energy is lower than the [Pr^3+^, M^4+^] energy at the configurational coordinates of the former system minimum; an oxidation of Pr^3+^ by M^4+^ is thus possible. This situation resembles a stable trap case, where initial (pretrapping) [Pr^3+^, M^4+^] states do not lie below the trapped electron [Pr^4+^, M^3+^] minimum.^25^ However, the activated [Pr^4+^, M^3+^] pair (the trapped electron system) minimal energy is lower than that of the [Pr^3+^, M^4+^] minimum energy. The [Pr^3+^, M^4+^] pair is thus not stable, while the [Pr^4+^, M^3+^] pair is fully stable (a thermodynamical minimum, not metastable)—the latter should not be considered a trap state. Pr^3+^ would be oxidized by M^4+^, provided that both are present in the sites corresponding to the described situation. This is always the case when Pr is in the C_3*i*_ site and the M codopant is in the C_2_ site (see Table 1). In the thermoluminescence context, the initial state is [Pr^3+^, M^4+^] (uncharged, empty trap), and the transition to [Pr^4+^, M^3+^] (charged, filled trap) happens upon irradiation by either ultraviolet or ionizing radiation. If the material is in the [Pr^4+^, M^3+^] state before the irradiation, the charging by irradiation cannot occur. Thus, the [, ] pair cannot correspond to postirradiation electron trapping as Pr is already ionized to Pr^4+^, while the M codopant is already 3+. On the other hand, the reverse [Pr^4+^, M^3+^] → [Pr^3+^, M^4+^] process is still possible and can be characterized by the trap depth, which, in this case, is quite high: about 3.5 eV for Ti, 3 eV for Zr and 2–2.5 eV for Hf (, ).

In the case of the Ti codopant, we do not observe the expected experimental trap depth of 1.8–2.5 eV.^5,6,11,19^ The calculated depth is either very low (0.033 eV Ti C_2_ and 0.91 Ti C_3*i*_; Pr C_2_), or the quench barriers are almost 0 eV (both Pr and Ti in the C_2_ sites, or both ions in the C_3*i*_ sites). The [Ti C_2_, Pr C_3*i*_] system exhibits unidirectional oxidation and a large trap depth (3.5 eV). Thus, Ti either does not work as an electron trap in Lu_2_O_3_ or (which is more likely) the detrapping mechanism must involve a conduction band. With the latter, the effect of quenching processes described in this work can be mitigated via an increase of the dopant–dopant distance—which is in line with the experimental low dopant concentrations (e.g., Kulesza et al.^5^).

Table 1 includes two sets of data for Hf. The two sets correspond to two kinds of Hf pseudopotentials used in the DFT geometry optimization (see the DFT Calculations subsection). Albeit different in values, the two sets result in the same conclusions. In particular, according to the results presented here, Hf does not correspond to the experimentally observed trapping in Lu_2_O_3_:Ln,Hf. Either the trap depths (TDs) are negligible ([Hf C_3*i*_, Pr C_3*i*_] and [Hf C_2_, Pr C_2_], 0.005–0.264 eV), the quench barriers (QBs) are low ([Hf C_2_, Pr C_3*i*_]), or the traps are too deep ([Hf C_3*i*_, Pr C_2_], 4–4.5 eV against the experimental 1.36 eV/1.44 eV^18,11^). Vacuum ultraviolet (120–130 nm) is required to populate the respective too-deep traps (OTPG), while the experimental thermoluminescence emission is observed after 250–320 nm irradiation.^5,10^ A noteworthy work of Kulesza et al. from 2022 presents a rare experiment on the thermoluminescence excitation spectrum,^19^ which indicates a distinct and relatively narrow band at 340–380 nm, as well as a broad multiband feature in the 250–330 nm range; all of these are attributed to Tb^3+^ f–d absorption.

The experimental dopant concentrations are very low, which likely corresponds to the low probabilities of the MMCT quenching processes. The [Hf C_2_, Pr C_3*i*_] is characterized by the trap depths of 2.0–2.5 eV (^1^D_2_, Table 1), which is rather close to the experimental value of about 1.36 eV/1.44 eV.^18,11^ This result is in line with the diabatic surface intersections providing “upper limit” estimates of the trap depth.

Similarly to the Hf codopant, in the case of the Zr codopant, the same pattern is observed in the dependence of the energetic parameters (trap depths, quench barriers, optical and quenching gaps) on the site symmetries. The trap depths are too high (with respect to the experimental value, 1.44 eV^11^) or the quench barriers are low. An interesting and noteworthy fact is that the calculated depths of the Zr-based traps are noticeably different from the respective values for Hf (Table 1). The differences for the deep trap depths are in the range of 0.5–1.5 eV for [Zr/Hf C_2_, Pr C_3*i*_] and [Zr/Hf C_3*i*_, Pr C_2_] systems. Such differences should be clearly distinguishable from the thermoluminescence glow curves. However, Sójka et al. indicate^11^ that Zr and Hf codopants result in identical glow curves in Lu_2_O_3_:Tb,M (M is Zr or Hf). According to the results presented here, the glow curves in the mentioned paper are unlikely to correspond to the [Pr^4+^, M^3+^] → [ ^1^D_2_ Pr^3+^, M^4+^] electron trapping and release mechanism—at least without the participation of a conduction band. If the mechanism was such, the trap depths and glow curves would not have been identical. This conclusion is quite important, and it can be argued that the pseudopotentials used to obtain the geometries might be a source of an error of some sort. We have consequently performed a calculation on the Zr dopant in the Hf geometry. Yet again, distinctly different trap depths were obtained, indicating that the difference originates from the Zr and Hf physical-chemical properties and not solely from the site geometries. The two elements are clearly not identical in their M^3+^–M^4+^ transitions. It is hence reasonable to expect that with the conduction band participation, Zr and Hf would also exhibit different energetics.

We would like to point out that the presented calculations correspond to the electron trapping and detrapping that do not involve a conduction band. The conduction band cases can be modeled using the same approach with Lu^3+^/Lu^2+^ instead of Pr. That problem, however, is more complex (in several aspects) and deserves a dedicated paper.

### Configuration Diagrams

**Figure 3:**
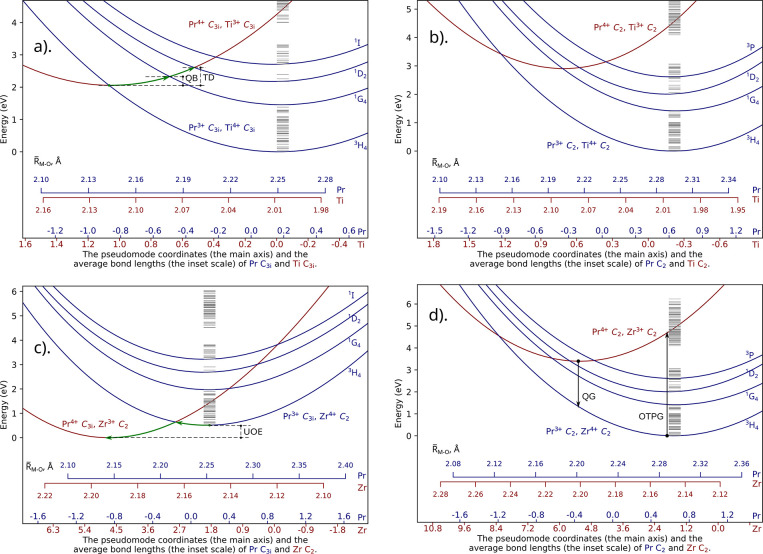
Selected configurational diagrams—the potential energy surface minimum-to-intersection lines as viewed along the Pr reaction coordinate/average bond length axis. The examples of the barriers summarized in Table 1 are provided. Note that in panel (a), the red curve minimum almost overlaps with another curve, and the minimal QB is close to zero. The calculated RASSI-SO Pr^3+^ levels (in the corresponding sites) are shown as gray dashes.

The data in Table 1 can be visualized using three-dimensional plots (Figure 2). However, such 3D plots are only readable and clear with two surfaces and one intersection line. Adding more surfaces makes the plot overwhelmingly complex. Additionally, a traditional way to visualize transitions is configurational diagrams (Figure 3), which are two-dimensional. Constructing the 2D diagrams from the 3D plots in question has its challenges. The points of interest in the 3D plots are the surfaces minima (*E*_*I*_ and *E*_*II*_ minima) and the minimum of the surface intersection line (Figure 2). A transition from the surface minimum (e.g., *E*_*I*_ minimum) to the *E*_*I*_/*E*_*II*_ surface-surface intersection minimum can take different paths that lie on the surface (e.g., *E*_*I*_). The energy difference between these two points is the barrier energy, no matter the path. To visualize the transition, we do not need to know the exact path as well. Thus, for each surface, we have chosen a path produced by the intersection of that surface by a vertical plane that contains both the surface minimum and the *E*_*I*_/*E*_*II*_ surface-surface intersection line minimum. This vertical plane is taken as the 2D graph (image) plane, and the respective path is the configurational diagram in the graph.

In the studied ion pairs, for every elementary MMCT, there is always the charged and uncharged state. Those are, for example, [Pr^4+^, M^3+^] (charged, filled trap, *E*_*I*_, the red surface in Figure 2) and [Pr^3+^, M^4+^] (uncharged, empty trap, *E*_*II*_, the blue surface in Figure 2). For two surfaces, there is a minimum for each and one intersection minimum (Figure 2). These three points do not have to lie in the same vertical plane—in our results, they do not. Albeit the intersection points are shared for the charged and uncharged states, individual vertical planes and individual paths are required for each surface. Moreover, we have considered several states of Pr^3+^. For a particular pair of Pr and codopant in the sites of particular symmetries, there is a separate surface corresponding to each electronic state of Pr^3+^. Accordingly, for each dopant pair and site symmetry, we have independently constructed the individual transition paths and put them on the same 3D plot. The diagrams in the subplots of Figure 3 are produced as side views of such 3D plots. The energy axis and the Pr average bond length (and pseudomode coordinate) axis are in the screen (2D graph) plane, and the codopant (Ti/Zr/Hf) geometry axis is perpendicular to the screen plane. Basically, for an individual 3D curve in the form of  data columns of the same length, column  was plotted against column *u*_Pr_. The one-to-one correspondence of the data was provided by the vertical cut of the surface.

The resulting plots are shown in Figure 3. The selected dopant, codopant and site symmetry combinations are shown. For the C_2_ Pr and C_3*i*_ M, the charged state (trapped electron) curves lie much higher than the Pr^3+^, M^4+^ curves. In the C_3*i*_ Pr and C_2_ M, the trapped electron curve minima lie below the Pr^3+^, M^4+^ minima. The Hf cases are qualitatively very similar to the respective Zr cases for the shown curves. From the curves shown in Figure 3, it is clear that the Ti traps are much deeper than the Zr traps. At the same time, quench barriers are clearly low, while vertical relaxation processes are possible. This confirms the previously formulated conclusions about the traps being prone to quenching in the Lu_2_O_3_:Pr,M(IV) systems (Table 1 and its description).

### Intervalence Charge Transfer: Carrier Migration

The previous section describes electron transfers that are traditionally considered in the context of thermoluminescence in Lu_2_O_3_ materials, namely, electron trapping on a codopant (e.g., ref (5)) . Another possibility is intervalence charge transfer (IVCT),^64^ where an electron is transferred between the ions of the same chemical element that differ in oxidation state. With the site symmetries in Lu_2_O_3_, those would be
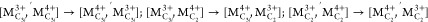


Table 2 summarizes the energetics of the M–M IVCTs in question. When the 3+ ion (donor) and the 4+ ion (acceptor) are of the same site symmetry, the electron transfer barriers are quite low, in the 0.1–0.5 eV range. This indicates that the trapped electrons can easily migrate among identical sites at temperatures much lower than room temperature. This property is another phenomenon related to the very low dopant concentrations required to achieve efficient energy storage in Pr-doped lutetium oxide.^3^

**Table 2:** Thermal Activation Barriers for M^4+^, ′M^3+^ → M^3+^, ′M^4+^ Intervalence Charge Transfer between Ti, Zr, and Hf Cations in c-Lu_2_O_3_

Site 1	Site 2	1 → 2 barrier (eV)	2 → 1 barrier (eV)
Ti C_3*i*_	Ti C_3*i*_	0.49	0.49
Ti C_3*i*_	Ti C_2_	0.27	3.33
Ti C_2_	Ti C_2_	0.34	0.34
Zr C_3*i*_	Zr C_3*i*_	0.38	0.38
Zr C_3*i*_	Zr C_2_	2.54	6.58
Zr C_2_	Zr C_2_	0.21	0.21
Hf^a^ C_3*i*_	Hf^a^ C_3*i*_	0.37	0.37
Hf^a^ C_3*i*_	Hf^a^ C_2_	2.24	6.39
Hf^a^ C_2_	Hf^a^ C_2_	0.16	0.16
Hf^b^ C_3*i*_	Hf^b^ C_3*i*_	0.43	0.43
Hf^b^ C_3*i*_	Hf^b^ C_2_	2.80	7.10
Hf^b^ C_2_	Hf^b^ C_2_	0.18	0.18

a Geometries obtained with Hf^4+^ pseudopotential.

b Geometries obtained with Hf^0^ pseudopotential.

On the contrary, when the site symmetries are different, there is a preferred direction of the intervalence charge transfer. In the case of Ti, the  process is characterized by a low barrier of 0.27 eV, while the reverse process barrier is 3.33 eV. Thus, the electrons trapped as  are likely to be irreversibly thermalized into . As previously stated, the  site corresponds to either a shallow (0.033 eV, Pr C_2_) or very deep trap (3.46 eV, Pr C_3*i*_), depending on the symmetry of the Pr site (Table 1 and the respective discussion). Similarly to Ti, IVCTs have a preferred direction for Zr and Hf as well—albeit the barriers are about 2.2–2.8 eV for  (M is Zr or Hf) and 6–7 eV for the reverse processes. As the lower of the two barriers is still quite high, the electron transfer between Zr or Hf ions of different site symmetries will not occur at room temperature. Comparing the Hf–Hf barriers to the Zr–Zr barriers in the respective site symmetries, we can observe that the values are similar but not identical. This supports the previous conclusion of Zr and Hf exhibiting different trap depths (at the end of Table 1 discussion).

**Figure 4:**
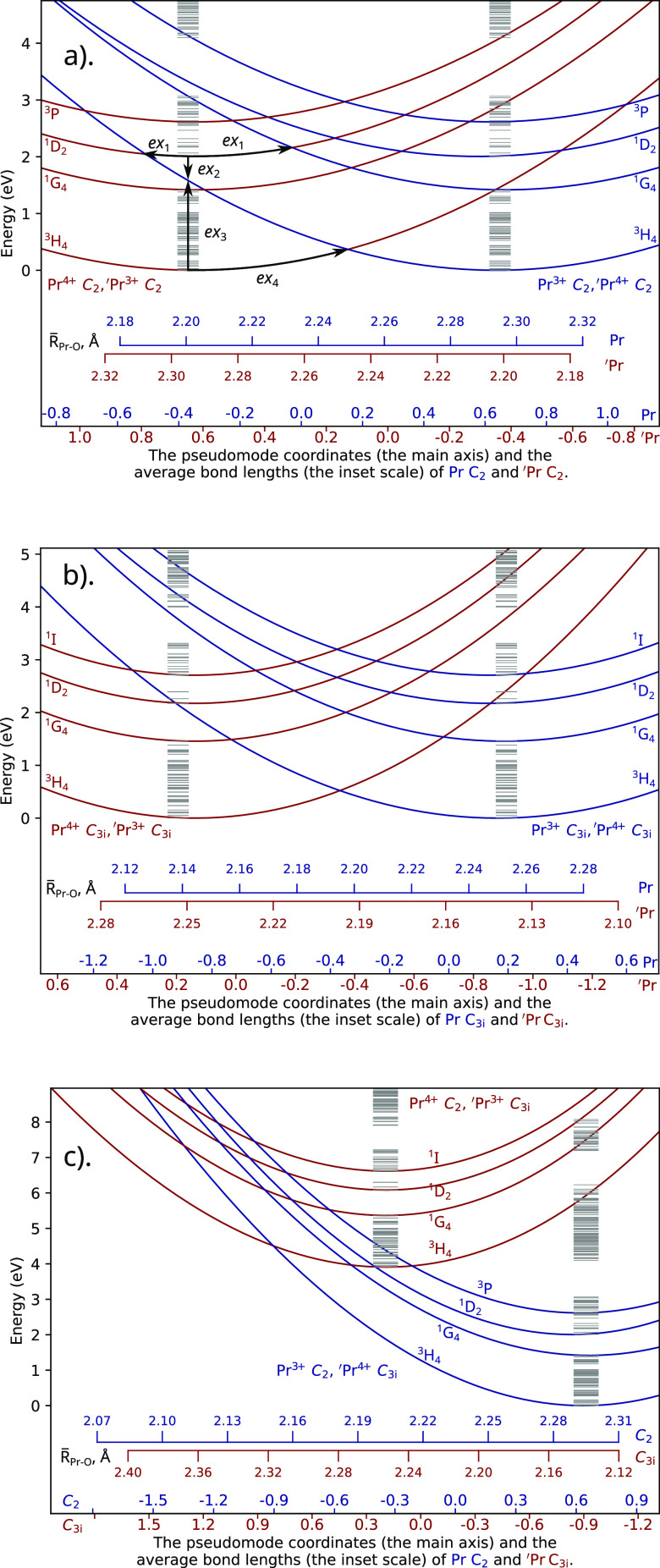
Configuration diagrams for the [Pr^3+^, ′Pr^4+^] ↔ [Pr^4+^, ′Pr^3+^] IVCT transitions. The examples of the thermal (TB) and optical (OB) barriers shown in subplot (a) are *ex*_1_: quenching (TB); *ex*_2_: relaxation (OB); *ex*_3_: excitation (OB); *ex*_4_: electron migration (TB). The calculated RASSI-SO Pr^3+^ levels (in the corresponding sites) are shown as gray dashes.

The same intervalence charge transfer analysis has been applied to Pr. However, excited states were considered in that case, resulting in a more complex map of possible transitions. The analyzed processes concern an electron transfer between a certain Pr ion and another ′Pr ion. The cases where both Pr and ′Pr ions are of the same site symmetry are shown in Figure 4(a) and (b). Similarly to the case of the M dopants, for both the  and  processes, the transitions involving the same excited state of Pr^3+^ on both sides were characterized by rather small thermalization barriers in the range of 0.3–0.5 eV (Table 3). For the same-symmetry processes, a [^3^H_4_ Pr^3+^, ′Pr^4+^] system can be thermally converted into either of the [Pr^4+^, ^1^D_2_ ′Pr^3+^], [Pr^4+^, ^3^P ′Pr^3+^], [Pr^4+^, ^1^I ′Pr^3+^] systems (i.e., the IVCT from the ground state of the donor can result in an excited state of the acceptor). Such an IVCT excitation would take about 2.0–2.2 eV to promote the acceptor Pr ion to its ^1^D_2_ state and about 2.8–2.9 eV to promote the acceptor Pr ion to the ^1^I/^3^P excited manifold. The former values correspond very well with the trap depths observed in Lu_2_O_3_:Pr without a codopant.^3^ That is, given the facts that the red thermoluminescence emission in Lu_2_O_3_:Pr,M corresponds to a Pr^3+1^D_2_ → ^3^H_4_ transition and that Pr^4+^ is present in Lu_2_O_3_:Pr, is it possible that stable Pr^4+^ in Lu_2_O_3_:Pr can act as an electron trap of 2.0–2.2 eV depth.

**Table 3:** Energy Barriers (Thermal Barriers (TB, eV) and Vertical/Optical Barriers (OB, eV)) of Electron Transfer Processes Involving Two Pr Ions for the Selected Pr^3+^ Levels^a^

Pr^3+^		′Pr^3+^		→ TB, OB (eV)		← TB, OB (eV)	
^3^H_4_	C_3i_	^3^H_4_	C_3i_	0.52	2.08	0.52	2.08
^1^G_4_	C_3i_	^1^G_4_	C_3i_	0.55	2.18	0.55	2.18
^1^D_2_	C_3i_	^1^D_2_	C_3i_	0.49	1.97	0.49	1.97
^1^I	C_3i_	^1^I	C_3i_	0.49	1.96	0.49	1.96
^3^H_4_	C_3i_	^1^G_4_	C_3i_	1.51	3.59	0.05	0.67
^3^H_4_	C_3i_	^1^D_2_	C_3i_	2.18	4.19	0.01	–0.15
^3^H_4_	C_3i_	^1^I	C_3i_	2.77	4.73	0.06	–0.69
^1^G_4_	C_3i_	^1^D_2_	C_3i_	0.94	2.78	0.22	1.36
^1^G_4_	C_3i_	^1^I	C_3i_	1.33	3.31	0.08	0.82
^1^D_2_	C_3i_	^1^I	C_3i_	0.79	2.50	0.26	1.42
^3^H_4_	C_2_	^3^H_4_	C_2_	0.37	1.47	0.37	1.47
^1^G_4_	C_2_	^1^G_4_	C_2_	0.39	1.55	0.39	1.55
^1^D_2_	C_2_	^1^D_2_	C_2_	0.32	1.29	0.32	1.29
^3^P	C_2_	^3^P	C_2_	0.36	1.42	0.36	1.42
^3^H_4_	C_2_	^1^G_4_	C_2_	1.42	2.93	0.00	0.09
^3^H_4_	C_2_	^1^D_2_	C_2_	2.07	3.38	0.07	–0.62
^3^H_4_	C_2_	^3^P	C_2_	2.86	4.06	0.24	–1.17
^1^G_4_	C_2_	^1^D_2_	C_2_	0.71	2.00	0.12	0.84
^1^G_4_	C_2_	^3^P	C_2_	1.21	2.68	0.01	0.29
^1^D_2_	C_2_	^3^P	C_2_	0.71	1.97	0.10	0.74
^3^H_4_	C_3i_	^3^H_4_	C_2_	0.60	–2.11	4.51	5.66
^1^G_4_	C_3i_	^3^H_4_	C_2_	1.65	–3.52	7.02	7.17
^1^D_2_	C_3i_	^3^H_4_	C_2_	2.57	–4.34	8.65	7.77
^1^I	C_3i_	^3^H_4_	C_2_	3.30	–4.88	9.91	8.30
^3^H_4_	C_3i_	^1^G_4_	C_2_	0.06	–0.65	2.55	4.28
^1^G_4_	C_3i_	^1^G_4_	C_2_	0.55	–2.06	4.50	5.79
^1^D_2_	C_3i_	^1^G_4_	C_2_	1.10	–2.87	5.77	6.39
^1^I	C_3i_	^1^G_4_	C_2_	1.57	–3.42	6.77	6.92
^3^H_4_	C_3i_	^1^D_2_	C_2_	0.01	–0.20	1.91	3.57
^1^G_4_	C_3i_	^1^D_2_	C_2_	0.37	–1.62	3.73	5.08
^1^D_2_	C_3i_	^1^D_2_	C_2_	0.87	–2.43	4.95	5.68
^1^I	C_3i_	^1^D_2_	C_2_	1.31	–2.97	5.92	6.21
^3^H_4_	C_3i_	^3^P	C_2_	0.03	0.48	1.33	3.017
^1^G_4_	C_3i_	^3^P	C_2_	0.12	–0.94	2.87	4.53
^1^D_2_	C_3i_	^3^P	C_2_	0.43	–1.75	3.90	5.13
^1^I	C_3i_	^3^P	C_2_	0.74	–2.29	4.74	5.66

a The [Pr^3+^, ′Pr^4+^] → [Pr^4+^, ′Pr^3+^] barriers are in the third column, and the reverse [Pr^3+^, ′Pr^4+^] ← [Pr^4+^, ′Pr^3+^] barriers are in the fourth column.

The reverse process (i.e., the intervalence charge transfer relaxation) can take numerous pathways originating from the numerous Pr^3+^ 4f levels—depending on the intersection positions between the states, as visualized in Figure 4. It is clear from Figure 4 that the [Pr^3+^, Pr^4+^] potential energy curves corresponding to Pr^3+^ in the lowest ^1^D_2_ level and the levels above intersect with the [Pr^4+^, ′Pr^3+^] potential energy curves corresponding to Pr^3+^ in some of its lower (nonemitting) levels. The barriers are low, and the respective quenching of Pr^3+^ emitting levels via IVCT to a Pr^4+^ should be efficient. This is one of the possible mechanisms for temperature-dependent cross-relaxation. However, as the excited states of lanthanides are usually long-lived, the quenching processes do not exclude a Pr^3+^ 4f–4f emission. As the barriers are different for the Pr ions of C_2_ and C_3*i*_ local symmetries (Table 3), there might be a difference in concentration quenching of the respective sites. Such an effect has been described by Bolek et al.^17^ for Tb, the dopant in the C_3*i*_ site being more prone to concentration quenching. From our calculations, the  process is characterized by a very small barrier, much lower than the respective barrier for the  process.

For the intervalence charge transfer processes, optical (vertical) transitions can be estimated as well. In particular, for the [^3^H_4_ Pr^3+^, ′Pr^4+^] → [Pr^4+^, ^3^H_4_ ′Pr^3+^] transition, the optical activation is 2.081 eV (595 nm) for the C_3*i*_ sites and 1.467 eV (845 nm) for the C_2_ sites. For the [^3^H_4_ Pr^3+^, ′Pr^4+^] → [Pr^4+^, ^1^D_2_ ′Pr^3+^] transition (excited ^1^D_2_ state formed at the acceptor), the optical activation is 4.192 eV (295.8 nm) for the C_3*i*_ sites and 3.375 ev (367.4 nm) for the C_2_ sites. The [^3^H_4_ Pr^3+^, ′Pr^4+^] → [Pr^4+^, ^1^I/^3^P ′Pr^3+^] transition can be excited with 4.723 eV (262.5 nm) for the C_3*i*_ sites (^1^I) and 4.055 eV (305.7 nm) for the C_2_ sites (^3^P). The two latter kinds of optical IVCT excitations should result in emission from the ^1^D_2_ manifold and correspond to the near-UV range, where broad excitation bands typically occur.^2^ Bands at similar positions are present in the excitation spectra of Lu_2_O_3_:Pr,Hf, as shown in the Ph.D. thesis of Aneta Wiatrowska,^65^ albeit the symmetries are assigned oppositely and the transition type is marked as an f–d band. From our calculations, the f–d transitions of Pr^3+^ in Lu_2_O_3_ start at about 300 nm and spread to higher energies: therefore, it is hard to attribute the band unambiguously to either the f–d or IVCT processes.

The values in Table 3 and Table 2 also provide another clue for why the dopant concentrations required for efficient thermoluminescence^3^ are usually low. The barriers for intervalence hopping of the electron from site to site are low, meaning that the electron is likely to either avoid recombination (an M–M process is more probable than an M–Pr process) or find a quenching center.

A much more complex pattern is observed if the local symmetries of the Pr sites are different. The  process is characterized by a large energy barrier of 4.51 eV, while the reverse process barrier is only 0.60 eV. At room temperature, the Pr^4+^ C_2_ sites are, therefore, likely to oxidize the Pr^3+^ C_3i_ sites. Such oxidation should also be expected from the fact that the  potential energy curve lies above the  one (Figure 4). Note that both Pr^3+^ and Pr^4+^ are present in Lu_2_O_3_,^9^ and, given our results, the 3+ sites should preferably be the C_2_ sites. This also agrees with the results presented by Kulesza et al.,^5^ where the emission in Lu_2_O_3_:Pr was observed from Pr C_2_ sites only.

If the  gets excited to its ^1^D_2_ state, the  process is characterized by a rather high barrier of 1.91 eV. If the  gets excited to its ^3^P state, the  process is characterized by a moderate barrier of 1.33 eV, meaning that, at room temperature, some  might be formed from the optically excited ^3^P  (assuming the presence of stable  prior to the excitation). Optical excitation at 219 nm/5.66 eV (or shorter waves/higher energies) might lead to the following intervalence charge transfer absorption: [, ^3^H_4_ (or higher) ] ( in its ground state). The  relaxation barrier to ^3^P  is only 0.032 eV, while the respective barrier of the relaxation to ^1^D_2_ is 0.006 eV, meaning that the mentioned intervalence charge transfer excitation will likely lead to the C_2_ Pr^3+^ emission anyway (see Figure 4 and Table 3). The described  would accordingly provide additional (likely broad) absorption/excitation bands in blue and near-UV—at the wavelengths typically used in the Lu_2_O_3_ excitation for thermoluminescence (250–320 nm;^10^ our result might overestimate the transition energies). With the presented results, we emphasize that IVCT absorption in doped Lu_2_O_3_ is expected to lie at about the same range of energies as the lanthanide f–d absorption. The f–d absorption corresponds to allowed transitions, while the IVCT absorption is expected to have lower intensity;^64^ the former is hence more likely to be dominant.

## Conclusions

In this paper, we have used DFT-derived geometries of metal clusters to construct a vibrational pseusomode coordination path, providing a vibration-like transformation between the coordination geometries of ions at different oxidation states. The paths were used as configurational coordinates to construct potential energy surfaces of ion cluster pairs and estimate metal-to-metal and intervalence charge transfer (MMCT and IVCT) energetics for selected dopants in Lu_2_O_3_, namely, Pr, Ti, Zr and Hf. The presented approach can be used with any kind of crystal and site symmetries.

For the material of interest, namely, Lu_2_O_3_:Pr,M (where M is Ti, Zr, Hf), Pr–Pr and M–M IVCTs, as well as Pr–M MMCTs were analyzed in the context of electron trapping and detrapping in addition to M trap state and Pr excited state quenching. The results indicate that [Pr^3+^, M^4+^] → [Pr^4+^, M^3+^] electron trapping is possible in principle, but the [Pr^4+^, M^3+^] filled trap state is not stable. There are many pathways that might result in the trapped electron release without the formation of Pr^3+^ excited states. Such a result explains the very small dopant concentrations (in the order of 0.01–0.1% mol) required to achieve efficient energy storage and thermoluminescence in Lu_2_O_3_:Pr,M: the dopants and codopants are efficient at quenching each other.

As for the particular values of the trap depths, the calculated MMCT trap depths values do not correspond well to the experimental values from the previous topical publications. This is not surprising, as the experimental glow curves are interpreted with the mechanisms involving electron transfers via a conduction band. Our results do not exclude other MMCT detrapping mechanisms that do not involve the conduction band: more defects and dopant coordination geometries can be involved. Our model does not give a trap depth with respect to the conduction band, but it clearly describes the optical charging and relaxation of the trap states as well as their quenching.

Another important conclusion regards similarities and differences in the trap depths and other barriers for the processes that involve Zr and Hf codopants. According to the previously published experimental data, Zr and Hf codoping corresponds to identical glow curves in Lu_2_O_3_:Ln,M.^11^ In the presented results, the Zr ions exhibit a pattern in the barrier values for various processes similar to that of the Hf ions—the particular values, however, are distinctly different for the two. In other words, according to the presented calculations, Zr and Hf cannot result in identical trap parameters. The two elements are clearly not identical in their M^3+^–M^4+^ transitions. Consequently, local conversion of Zr/Hf ions from 4+ to 3+ is unlikely to be the electron trapping mechanism with those codopants. This conclusion is also in line with the DFT results for Hf in Lu_2_O_3_.^66^

The Pr–Pr intervalence charge transfer might play an important role in the electron trapping and emission properties of Lu_2_O_3_:Pr. In particular, our results indicate that Pr^3+^ ions are less stable in C_3i_ sites than in the C_2_ sites. If Pr in one of the sites had to be 4+, that would rather be the C_3i_ site. An IVCT process of the excited acceptor formation—[^3^H_4_ Pr^3+^, ′Pr^4+^] → [Pr^4+^,^1^D_2_ ′Pr^3+^]—is characterized by the activation barrier of 2.0–2.2 eV (if both ions occupy sites of the same symmetry) and can hence be an explanation for the 2 eV electron trap observed experimentally in Lu_2_O_3_:Pr.^3^

## Supporting Information Available

Two Python scripts and a set of files with total energies that can be used to construct the surfaces and the configuration diagrams, including instructions on how to use the scripts (ZIP)
DFT calculation details, cluster geometries, embedding AIMP files, and configuration diagrams for all Pr–M pairs (PDF)
