# Supplementary material for: Electron Trap Depths in Cubic Lutetium Oxide Doped with Pr and Ti, Zr or Hf—From Ab Initio Multiconfigurational Calculations
Source: J Phys Chem A. 2023 May 17;127(21):4583–95. doi: 10.1021/acs.jpca.2c07979 (PMC10240500; doi:10.1021/acs.jpca.2c07979)
Supplement: Supplementary file 2 — jp2c07979_si_002.pdf [file jp2c07979_si_002.pdf]

# Electron Trap Depths in Cubic Lutetium Oxide Doped with Pr and Ti, Zr or Hf – from A*b Initio* Multiconfigurational Calculations

Andrii Shyichuk*,† and Marek Krośnicki*,‡

†Faculty of Chemistry, University of Wroclaw, ul. F. Joliot-Curie 14, 50-383 Wroclaw, Poland
‡Institute of Theoretical Physics and Astrophysics, Faculty of Mathematics, Physics and Informatics, University of Gdańsk, ul. Wita Stwosza 57, 80-308 Gdańsk, Poland
E-mail: andrii.shyichuk@chem.uni.wroc.pl; marek.krosnicki@ug.edu.pl

## Supplementary Information

### DFT calculations

Initial geometry (in the from of fractional coordinates of atoms) was generated by Spacegroup code shipped with Elk code version 4.3.6 (*http://elk.sourceforge.net/*), using space group Ia-3, all cell angles of 90.0 degrees and the following fractional coordinates of atoms (*10.3390/ma7107059*):

Lu1: 0.25, 0.25, 0.25;  Lu2: 0.46685, 0.0, 0.25;  O: 0.39159, 0.15282, 0.38011

Positions of the rest of the atoms were generated by the symmetry operations respective to the space group. Note that Lu1 and Lu2 here are site labels, not to be confused with the atom labels used below. The following input for Spacegroup was used:

```
'Ia-3'
19.6403 19.6403 19.6403
90.0   90.0   90.0
1 1 1
.false.
2
'Lu'
2
0.25    0.25   0.25
0.46685 0.0    0.25
'O'
1
0.39159 0.15282 0.38011
```

The dopants were put in either $C_{3i}$ or $C_2$ Lu site. For each dopant and site, a separate input was created. The cells were translated so that the dopant ion was at the origin in fractional (crystal) coordinates. The geometries were optimized with Quantum Espresso 6.5 (*https://www.quantum-espresso.org/*, *10.1088/1361-648X/aa8f79*, *10.1088/0953-8984/21/39/395502*). Module pw.x (total energy calculations and geometry optimization) was used with mostly default settings. PBEsol generalized gradient approximation functional (*10.1103/PhysRevLett.100.136406*) was used. Crucial settings are shown in Table S1.

The pseudopotentials (PPs) used were ultrasoft (USPP), generated by the Vanderbilt code version 7.3.6. The PP recipes (PP generation input files) for O, Ti, Zi, Hf from GBRV group (http://www.physics.rutgers.edu/gbrv/, *10.1016/j.commatsci.2013.08.053*) were used. The Lu USPP recipe was kindly provided by co-author of the GBRV set, Kevin F. Garrity. The potential had the 4f electrons in core; the valence electrons were effectively of a *d*-element, not an *f*-element. The Vanderbilt code was modified to support PBEsol functional. The Pr PP was a projector augmented wave (PAW) PP by Topsakal and Wentzcovitch (TW PAW) (*10.1103/PhysRevLett.100.136406*, http://www.mineralscloud.com/resources/repaw/index.shtml), used without +U. Both LDA and PBE Pr PPs were tested, as the PBEsol one was not available. The differences in geometries were negligible, and the PBE version was used with PBEsol functional and PBEsol PPs for the rest of the system.

Table S1. Selected settings used in Quantum Espresso calculations.

| Section &CONTROL | |
|---|---|
| calculation | 'vc-relax' |
| etot_conv_thr | 1E-5 |
| forc_conv_thr | 0.0001 |
| nstep | 700 |

| Section &SYSTEM | |
|---|---|
| ecutwfc | 40 |
| ecutrho | 400 |
| occupations | 'smearing' |
| smearing | 'gaussian' |
| degauss | 0.001 |
| nspin | 1 or 2 * |
| tot_charge | 1 / 0 * |

| Section &ELECTRONS | |
|---|---|
| electron_maxstep | 200 |
| conv_thr | 1.0D-7 |
| diago_thr_init | 1.0E-4 |
| startingpot | 'atomic' |
| mixing_mode | 'plain' |
| mixing_beta | 0.5 |
| mixing_ndim | 8 |
| diagonalization | 'david' |

| Section &IONS | |
|---|---|
| ion_dynamics | 'bfgs' |

| Section &CELL | |
|---|---|
| cell_dynamics | 'bfgs' |

* tot_charge is total charge on the system, while nspin controls spin polarization (1 for unpolarized and 2 for polarized); spin-polarization was used for systems with odd tot_charge.

## Atom surround geometries as obtained by QE/PBEsol

The coordination geometries of the atoms from the density functional theory (DFT) calculations (QE/PBEsol as described above) are shown in the following table. During optimization, the initial site symmetries ($C_{3i}$ and $C_2$) were maintained in both the initial and the final geometries. In each of the geometries below, the positions of the second group of three oxygens are symmetric to the first group of three oxygens either by inversion ($C_i$, or XYZ in MOLCAS notation) or a 180 degree rotation around *x* axis ($C_{2\text{-}x}$, or YZ in MOLCAS notation). The $C_{3i}$ sites had $C_i$ symmetry in MOLCAS calculations due to the fact that MOLCAS does not have the respective three-fold rotation symmetry generator. The Cartesian displacements for the pseudomodes can be obtained as element-vise (matrix-matrix) difference between any of the two geometries.

Table S2. Coordination geometries of the dopants obtained using DFT and used in the MOLCAS calculations, as described in the main text

| Sites of the $C_{3i}$ / $C_i$ / XYZ symmetry | | | | | $\bar{R}_{M\text{-}O}$, Å | Sites of the $C_2$ / $C_{2\text{-}x}$ / YZ symmetry | | | | | $\bar{R}_{M\text{-}O}$, Å | M, PP |
|---|---|---|---|---|---|---|---|---|---|---|---|---|
| No. | Label | X | Y | Z | | No. | Label | X | Y | Z | | |
| 1 | PR_1 | 0.000000 | 0.000000 | 0.000000 | | 1 | PR_2 | 0.000000 | 0.000000 | 0.000000 | | |
| 2 | O_01 | 1.462962 | -1.025768 | 1.338223 | | 2 | O_02 | -0.799741 | 1.592904 | 1.350495 | | |
| 3 | O_02 | -1.025768 | 1.338223 | 1.462962 | | 3 | O_03 | 1.567118 | -1.134221 | 1.022607 | | |
| 4 | O_03 | -1.338223 | -1.462962 | 1.025768 | 2.2323 | 4 | O_04 | -1.245724 | -1.235708 | 1.450477 | 2.2336 | $Pr^{4+}$, $Pr^{3+}$ TW PAW |
| 5 | O_01 | -1.462962 | 1.025768 | -1.338223 | | 5 | O_02 | -0.799741 | -1.592904 | -1.350495 | | |
| 6 | O_02 | 1.025768 | -1.338223 | -1.462962 | | 6 | O_03 | 1.567118 | 1.134221 | -1.022607 | | |
| 7 | O_03 | 1.338223 | 1.462962 | -1.025768 | | 7 | O_04 | -1.245724 | 1.235708 | -1.450477 | | |
| 1 | PR_1 | 0.000000 | 0.000000 | 0.000000 | | 1 | PR_2 | 0.000000 | 0.000000 | 0.000000 | | |
| 2 | O_01 | 1.523788 | -1.099761 | 1.387881 | | 2 | O_02 | -0.904336 | 1.638656 | 1.380630 | | |
| 3 | O_02 | -1.099761 | 1.387881 | 1.523788 | | 3 | O_03 | 1.598945 | -1.195577 | 1.072296 | | |
| 4 | O_03 | -1.387881 | -1.523788 | 1.099761 | 2.3362 | 4 | O_04 | -1.305078 | -1.288957 | 1.529246 | 2.3267 | $Pr^{3+}$, TW PAW |
| 5 | O_01 | -1.523788 | 1.099761 | -1.387881 | | 5 | O_02 | -0.904336 | -1.638656 | -1.380630 | | |
| 6 | O_02 | 1.099761 | -1.387881 | -1.523788 | | 6 | O_03 | 1.598945 | 1.195577 | -1.072296 | | |
| 7 | O_03 | 1.387881 | 1.523788 | -1.099761 | | 7 | O_04 | -1.305078 | 1.288957 | -1.529246 | | |
| 1 | Ti_1 | 0.000000 | 0.000000 | 0.000000 | | 1 | TI_2 | 0.000000 | 0.000000 | 0.000000 | | |
| 2 | O_01 | 1.330981 | -0.828999 | 1.253888 | | 2 | O_02 | -0.613682 | 1.409311 | 1.233222 | | |
| 3 | O_02 | -0.828999 | 1.253888 | 1.330981 | | 3 | O_03 | 1.453306 | -0.996364 | 0.887843 | | |
| 4 | O_03 | -1.253888 | -1.330981 | 0.828999 | 2.0077 | 4 | O_04 | -1.167919 | -1.155647 | 1.340972 | 2.0215 | $Ti^{4+}$, Ti GBRV |
| 5 | O_01 | -1.330981 | 0.828999 | -1.253888 | | 5 | O_02 | -0.613682 | -1.409311 | -1.233222 | | |
| 6 | O_02 | 0.828999 | -1.253888 | -1.330981 | | 6 | O_03 | 1.453306 | 0.996364 | -0.887843 | | |
| 7 | O_03 | 1.253888 | 1.330981 | -0.828999 | | 7 | O_04 | -1.167919 | 1.155647 | -1.340972 | | |
| 1 | Ti_1 | 0.000000 | 0.000000 | 0.000000 | | 1 | TI_2 | 0.000000 | 0.000000 | 0.000000 | | |
| 2 | O_01 | 1.380056 | -0.933094 | 1.294285 | | 2 | O_02 | -0.620370 | 1.462521 | 1.271672 | | |
| 3 | O_02 | -0.933094 | 1.294285 | 1.380056 | | 3 | O_03 | 1.527584 | -1.061419 | 0.952764 | | |
| 4 | O_03 | -1.294285 | -1.380056 | 0.933094 | 2.1096 | 4 | O_04 | -1.195929 | -1.221598 | 1.427299 | 2.1173 | $Ti^{3+}$, Ti GBRV |
| 5 | O_01 | -1.380056 | 0.933094 | -1.294285 | | 5 | O_02 | -0.620370 | -1.462521 | -1.271672 | | |
| 6 | O_02 | 0.933094 | -1.294285 | -1.380056 | | 6 | O_03 | 1.527584 | 1.061419 | -0.952764 | | |
| 7 | O_03 | 1.294285 | 1.380056 | -0.933094 | | 7 | O_04 | -1.195929 | 1.221598 | -1.427299 | | |

| | | | | | | | | | | | | |
|---|---|---|---|---|---|---|---|---|---|---|---|---|
| 1 | ZR_1 | 0.000000 | 0.000000 | 0.000000 | | 1 | ZR_2 | 0.000000 | 0.000000 | 0.000000 | | |
| 2 | O_01 | 1.397540 | -0.915672 | 1.296102 | | 2 | O_02 | -0.687535 | 1.493702 | 1.286709 | | |
| 3 | O_02 | -0.915672 | 1.296102 | 1.397540 | | 3 | O_03 | 1.537675 | -1.058020 | 0.942284 | | |
| 4 | O_03 | -1.296102 | -1.397540 | 0.915672 | 2.1146 | 4 | O_04 | -1.175842 | -1.190106 | 1.394541 | 2.1189 | $Zr^{4+}$, Zr GBRV |
| 5 | O_01 | -1.397540 | 0.915672 | -1.296102 | | 5 | O_02 | -0.687535 | -1.493702 | -1.286709 | | |
| 6 | O_02 | 0.915672 | -1.296102 | -1.397540 | | 6 | O_03 | 1.537675 | 1.058020 | -0.942284 | | |
| 7 | O_03 | 1.296102 | 1.397540 | -0.915672 | | 7 | O_04 | -1.175842 | 1.190106 | -1.394541 | | |
| 1 | ZR_1 | 0.000000 | 0.000000 | 0.000000 | | 1 | ZR_2 | 0.000000 | 0.000000 | 0.000000 | | |
| 2 | O_01 | 1.416818 | -1.016070 | 1.314071 | | 2 | O_02 | -0.714607 | 1.505634 | 1.290553 | | |
| 3 | O_02 | -1.016070 | 1.314071 | 1.416818 | | 3 | O_03 | 1.533645 | -1.082396 | 0.932627 | | |
| 4 | O_03 | -1.314071 | -1.416818 | 1.016070 | 2.1832 | 4 | O_04 | -1.196910 | -1.196504 | 1.399024 | 2.1332 | $Zr^{3+}$, Zr GBRV |
| 5 | O_01 | -1.416818 | 1.016070 | -1.314071 | | 5 | O_02 | -0.714607 | -1.505634 | -1.290553 | | |
| 6 | O_02 | 1.016070 | -1.314071 | -1.416818 | | 6 | O_03 | 1.533645 | 1.082396 | -0.932627 | | |
| 7 | O_03 | 1.314071 | 1.416818 | -1.016070 | | 7 | O_04 | -1.196910 | 1.196504 | -1.399024 | | |
| 1 | HF_1 | 0.000000 | 0.000000 | 0.000000 | | 1 | HF_2 | 0.000000 | 0.000000 | 0.000000 | | |
| 2 | O_01 | 1.388573 | -0.901466 | 1.290721 | | 2 | O_02 | -0.671406 | 1.486029 | 1.283818 | | |
| 3 | O_02 | -0.901466 | 1.290721 | 1.388573 | | 3 | O_03 | 1.530349 | -1.048621 | 0.935390 | | |
| 4 | O_03 | -1.290721 | -1.388573 | 0.901466 | 2.0992 | 4 | O_04 | -1.169084 | -1.180059 | 1.380303 | 2.1043 | $Hf^{4+}$, $Hf^0$ GBRV |
| 5 | O_01 | -1.388573 | 0.901466 | -1.290721 | | 5 | O_02 | -0.671406 | -1.486029 | -1.283818 | | |
| 6 | O_02 | 0.901466 | -1.290721 | -1.388573 | | 6 | O_03 | 1.530349 | 1.048621 | -0.935390 | | |
| 7 | O_03 | 1.290721 | 1.388573 | -0.901466 | | 7 | O_04 | -1.169084 | 1.180059 | -1.380303 | | |
| 1 | HF_1 | 0.000000 | 0.000000 | 0.000000 | | 1 | HF_2 | 0.000000 | 0.000000 | 0.000000 | | |
| 2 | O_01 | 1.389220 | -0.911733 | 1.298903 | | 2 | O_02 | -0.686155 | 1.486207 | 1.288938 | | |
| 3 | O_02 | -0.911733 | 1.298903 | 1.389220 | | 3 | O_03 | 1.528739 | -1.061025 | 0.937012 | | |
| 4 | O_03 | -1.298903 | -1.389220 | 0.911733 | 2.1091 | 4 | O_04 | -1.178436 | -1.185185 | 1.381250 | 2.1117 | $Hf^{3+}$, $Hf^0$ GBRV |
| 5 | O_01 | -1.389220 | 0.911733 | -1.298903 | | 5 | O_02 | -0.686155 | -1.486207 | -1.288938 | | |
| 6 | O_02 | 0.911733 | -1.298903 | -1.389220 | | 6 | O_03 | 1.528739 | 1.061025 | -0.937012 | | |
| 7 | O_03 | 1.298903 | 1.389220 | -0.911733 | | 7 | O_04 | -1.178436 | 1.185185 | -1.381250 | | |
| 1 | HF_1 | 0.000000 | 0.000000 | 0.000000 | | 1 | HF_2 | 0.000000 | 0.000000 | 0.000000 | | |
| 2 | O_01 | 1.417467 | -0.932192 | 1.308355 | | 2 | O_02 | -0.699719 | 1.516724 | 1.302725 | | |
| 3 | O_02 | -0.932192 | 1.308355 | 1.417467 | | 3 | O_03 | 1.558589 | -1.070677 | 0.954675 | | |
| 4 | O_03 | -1.308355 | -1.417467 | 0.932192 | 2.1424 | 4 | O_04 | -1.182648 | -1.200373 | 1.411238 | 2.1448 | $Hf^{4+}$, $Hf^{4+}$ GBRV |
| 5 | O_01 | -1.417467 | 0.932192 | -1.308355 | | 5 | O_02 | -0.699719 | -1.516724 | -1.302725 | | |
| 6 | O_02 | 0.932192 | -1.308355 | -1.417467 | | 6 | O_03 | 1.558589 | 1.070677 | -0.954675 | | |
| 7 | O_03 | 1.308355 | 1.417467 | -0.932192 | | 7 | O_04 | -1.182648 | 1.200373 | -1.411238 | | |
| 1 | HF_1 | 0.000000 | 0.000000 | 0.000000 | | 1 | HF_2 | 0.000000 | 0.000000 | 0.000000 | | |
| 2 | O_01 | 1.428181 | -1.027207 | 1.320653 | | 2 | O_02 | -0.722071 | 1.524426 | 1.305497 | | |
| 3 | O_02 | -1.027207 | 1.320653 | 1.428181 | | 3 | O_03 | 1.554432 | -1.090666 | 0.950610 | | |
| 4 | O_03 | -1.320653 | -1.428181 | 1.027207 | 2.1998 | 4 | O_04 | -1.198497 | -1.204820 | 1.411953 | 2.1553 | $Hf^{3+}$, $Hf^{4+}$ GBRV |
| 5 | O_01 | -1.428181 | 1.027207 | -1.320653 | | 5 | O_02 | -0.722071 | -1.524426 | -1.305497 | | |
| 6 | O_02 | 1.027207 | -1.320653 | -1.428181 | | 6 | O_03 | 1.554432 | 1.090666 | -0.950610 | | |
| 7 | O_03 | 1.320653 | 1.428181 | -1.027207 | | 7 | O_04 | -1.198497 | 1.204820 | -1.411953 | | |

**Embedding structure preparation**

In order to perform an ab initio model potential (AIMP) embedded cluster calculation, the embedding structure representing the lattice is required. The structure consists of the cluster atoms represented by a full atomic basis set, of the AIMPs around the cluster and point charges further on. The positions of the AIMPs and charges corresponded to the experimental $Lu_2O_3$ structure. The values of the point charges where -2 for O and +3 for Lu at proximity of the AIMP layer, and where reduced with the increasing distance from the cluster. The values of the charges and the amount of them were selected to keep the electric multipole moments (of order 1, 2 and 3) at the cluster as low as possible. That is the method by Alain Gellé and Marie-Bernadette Lepetit, implemented in Lattgen code (10.1063/1.2931458). The input for the code corresponded to the experimental crystal geometry, while the embedding control parameters are given in Table S3.

Table 3. Embedding control parameters for Lattgen conde

| Parameter and value | Meaning |
|---|---|
| dsys=3.0 | Cluster radius 3 Å |
| dpseud=12.0 | AIMP layer radius 12 Å |
| ncel=1 | The number of ionic cells surrounding the main unit cell that maintain their nominal charges. |
| single=T, ndip=3 | Require electric multipole moments (of order ≤3) to be zero. |

**Ab initio model potentials (AIMPs) for embedding**

Lu and O AIMPs were obtained using self-consistent embedding ion (SCEI) procedure, as described in 10.1142/9789812815156_0002. To make our results reproducible, we include the respective ready-to-use AIMPLIB file below. The file includes “regular” AIMPs for Lu and O, and the so-called orthogonalization AIMPs for Lu. The latter are AIMPs with orbital (basis set) components. Such AIMPs were used for Lu atoms that are in direct contact with the oxygens of the cluster – which are also the second coordination layer of the central (dopant) ions. The orbitals on these AIMPs do not get occupied, and ensure orthogonality between the cluster and the lattice (the embedding). Thanks to these orbitals, the cluster electrons expirience Pauli repulsion excerted by the embedding. The details are explained in 10.1063/1.464350.

```
* ==============================================================
* ===== Lu2O3 Ia-3 AIMPs Andrii Shyichuk  -   AIMPLIB file =====
* ==============================================================
*
*
Lu.ECP.Shyichuk.27s23p15d10f.1s1p1d1f.0e-AIMP-Lu2O3-L1.
Orthogonalization AIMP for Lu in C3i site
Structure: Zeler et al., Materials 7(2014)7059, 10.3390/ma7107059
   3.000    3
    27    1
 0.6701311900E+11  7216717800.      1171529200.      235233120.0
  55877724.00      14949778.00      4363017.000      1375340.500
  456540.5200      160435.3700      59637.10400      23233.14400
  9474.040900      4032.731300      1779.753900      807.8776800
  374.9260700      172.1622700      81.75905400      34.04620200
  17.67359800      7.084092600      3.479507300     0.9931652500
 0.4458321400     0.7721303500E-01 0.3053352200E-01
 -0.000000006602
  0.000000030374
 -0.000000110116
  0.000000467131
 -0.000019814395
 -0.000025438669
 -0.000065022341
 -0.000135455942
 -0.000276195204
 -0.000561989435
 -0.001128728096
 -0.002287802822
 -0.004591001203
 -0.009004931174
 -0.015776321530
 -0.019863918091
  0.003675558178
  0.095659912538
  0.150368486923
 -0.208811271663
 -0.476439144083
  0.433749447001
  0.774126811080
 -0.696902139902
 -0.667784548498
  0.018649282941
 -0.002574922135
   23 1
  55602773.00      7765745.900      1589584.300      401810.9500
  118603.3400      39300.00000      14430.35400      5793.124600
  2497.821500      1143.717300      551.3977100      277.7501400
  145.0303300      77.44745600      41.75015500      23.22694800
  12.93638700      7.026064900      3.744504700      1.899190800
 0.9146347400     0.4226736900     0.1733363600
 -0.000000211731
 -0.000002223711
 -0.000007456779
```

```
 -0.000016278231
 -0.000053035340
 -0.000139018757
 -0.000390410663
 -0.001088011353
 -0.003035086569
 -0.007929887039
 -0.018132873849
 -0.031999439549
 -0.034189488144
  0.013693235271
  0.111419748918
  0.156109722792
 -0.051078449881
 -0.274078472763
 -0.313953683951
  0.140504495696
  0.517074336971
  0.543180129564
 -0.015735095708
   15    1
  16241.49300      4278.988200      1524.597100      640.8577100
  295.5655200      143.5265800      71.89617500      36.65707500
  18.88423700      9.510456800      4.620822600      2.112008600
  0.7516843600     0.2514027000     0.7547191500E-01
 -0.000041407692
 -0.000295436661
 -0.001623867413
 -0.007256532095
 -0.026158801412
 -0.075632836866
 -0.152363359938
 -0.180756951869
  0.020915538014
  0.385953039661
  0.487110565565
  0.223638693255
  0.021457369777
 -0.001738535917
  0.000336961233
   10    1
  1573.111300      295.9291500      108.3458800      47.08844000
  21.82595400      10.48002400      4.988653200      2.304761500
 0.9995225700     0.3810215700
  0.000091140854
  0.002174076220
  0.015331997160
  0.061796932393
  0.166120843734
  0.288032305531
  0.343072997326
  0.296226933977
  0.188748779124
  0.061891705617
*
* External AIMP:
* Local Pot. Param. :
*                     A(AIMP)=-Zeff*A(ECP)
*
M1
   15
  573250.0000      124682.0000      38556.00000      13233.40000
  4895.600000      1863.700000      729.9500000      332.2300000
  125.6600000      59.61100000      29.10400000      10.59000000
  5.020600000      1.796200000     0.8441200000
 0.2012900221     0.1781175038     0.2895424193     0.4474200300
 0.6139239039     0.7686169723      1.306066160      1.768520444
  1.289621356      4.236266210     0.5430171971      5.140778014
  2.701068441      2.254929883     0.9274881105
M2
    0
```

```
COREREP
   1.000
PROJOP
    3
   27    5    2    2    2    2    2
  4672.119192      807.7374734      189.2668345      41.83658654
  7.894165093
 0.6701311900E+11  7216717800.      1171529200.      235233120.0
  55877724.00      14949778.00      4363017.000      1375340.500
  456540.5200      160435.3700      59637.10400      23233.14400
  9474.040900      4032.731300      1779.753900      807.8776800
  374.9260700      172.1622700      81.75905400      34.04620200
  17.67359800      7.084092600      3.479507300     0.9931652500
 0.4458321400     0.7721303500E-01 0.3053352200E-01
 -0.000000244159 -0.000000081509 -0.000000051623  0.000000015328 -0.000000006602
  0.000001120858  0.000000374559  0.000000236647 -0.000000070588  0.000000030374
 -0.000004011432 -0.000001348943 -0.000000839506  0.000000257506 -0.000000110116
  0.000016057858  0.000005559797  0.000003220047 -0.000001123109  0.000000467131
 -0.000595541247 -0.000221485142 -0.000106087807  0.000050449202 -0.000019814395
 -0.000742824063 -0.000280859243 -0.000128808383  0.000065473198 -0.000025438669
 -0.001956848975 -0.000727632218 -0.000351836447  0.000165070969 -0.000065022341
 -0.003949491597 -0.001497904726 -0.000690998486  0.000348558245 -0.000135455942
 -0.008181500877 -0.003073808998 -0.001481836453  0.000700415445 -0.000276195204
 -0.016327088540 -0.006257083642 -0.002933260892  0.001449252122 -0.000561989435
 -0.032284657317 -0.012495618809 -0.005959475717  0.002885488408 -0.001128728096
 -0.063239741794 -0.025221331465 -0.011988746597  0.005864833971 -0.002287802822
 -0.120490512185 -0.050090556902 -0.024128817125  0.011760042489 -0.004591001203
 -0.213402967907 -0.096618044489 -0.046861587674  0.023033930823 -0.009004931174
 -0.315171204704 -0.163495724269 -0.082167298278  0.040358698261 -0.015776321530
 -0.302931771382 -0.195815360358 -0.100636631151  0.050567030453 -0.019863918091
 -0.121186283481  0.010629428500  0.012943715203 -0.008841450911  0.003675558178
 -0.007602141998  0.503630591588  0.431201189209 -0.238268061028  0.095659912538
 -0.000655828604  0.534062026008  0.582640824847 -0.363876452546  0.150368486923
  0.000524010169  0.095859164291 -0.464931114345  0.457450390598 -0.208811271663
 -0.000298565536 -0.014771785989 -0.818865068909  0.986626606953 -0.476439144083
  0.000144199912  0.005047171875 -0.115528665973 -0.614292526307  0.433749447001
 -0.000076444751 -0.002355681607  0.014445926463 -0.857273926704  0.774126811080
  0.000025311211  0.000734669729 -0.003840156887 -0.057286720821 -0.696902139902
 -0.000011469543 -0.000343809841  0.001777728159  0.012691608588 -0.667784548498
  0.000002932694  0.000086247918 -0.000414561761 -0.002181867063  0.018649282941
 -0.000001429421 -0.000040360587  0.000197945719  0.001110176627 -0.002574922135
   23    4    6    6    6    6
  712.8494695      159.8678645      31.76916200      5.063535877
  55602773.00      7765745.900      1589584.300      401810.9500
  118603.3400      39300.00000      14430.35400      5793.124600
  2497.821500      1143.717300      551.3977100      277.7501400
  145.0303300      77.44745600      41.75015500      23.22694800
  12.93638700      7.026064900      3.744504700      1.899190800
 0.9146347400     0.4226736900     0.1733363600
  0.000001787485 -0.000000941647 -0.000000369676 -0.000000211731
  0.000029778262 -0.000014803267 -0.000007475672 -0.000002223711
  0.000077473637 -0.000039657055 -0.000017830965 -0.000007456779
  0.000219006573 -0.000109129009 -0.000055186956 -0.000016278231
  0.000590313060 -0.000299795562 -0.000141846684 -0.000053035340
  0.001617915777 -0.000820945151 -0.000396170308 -0.000139018757
  0.004448869540 -0.002272298527 -0.001092132632 -0.000390410663
  0.012330210985 -0.006350247575 -0.003064269999 -0.001088011353
  0.033632733937 -0.017577786075 -0.008499381949 -0.003035086569
  0.085361492404 -0.045767075842 -0.022290108595 -0.007929887039
  0.184634467298 -0.103019734929 -0.050574247850 -0.018132873849
  0.306178043485 -0.179921982031 -0.089738115133 -0.031999439549
  0.333940359115 -0.192209940929 -0.094505831349 -0.034189488144
  0.191850774038  0.015578030291  0.030481272923  0.013693235271
  0.041370581168  0.389528330318  0.292147926177  0.111419748918
  0.001846184747  0.478691276674  0.385608279752  0.156109722792
  0.000145653423  0.202680590145 -0.045159697777 -0.051078449881
 -0.000133664932  0.026110805231 -0.546223724282 -0.274078472763
 -0.000015766198  0.001456433841 -0.481882985622 -0.313953683951
 -0.000005991598  0.000167969926 -0.118359068355  0.140504495696
  0.000001323965 -0.000007282852 -0.003754261733  0.517074336971
  0.000000080299  0.000008479797 -0.001524517978  0.543180129564
```

```
 -0.000000146269 -0.000008211187  0.000618555432 -0.015735095708
   15    2   10   10
  123.6886766      18.53239027
  16241.49300      4278.988200      1524.597100      640.8577100
  295.5655200      143.5265800      71.89617500      36.65707500
  18.88423700      9.510456800      4.620822600      2.112008600
 0.7516843600     0.2514027000     0.7547191500E-01
  0.000085227076 -0.000041407692
  0.000604044322 -0.000295436661
  0.003323657658 -0.001623867413
  0.014681007630 -0.007256532095
  0.052573202795 -0.026158801412
  0.148494365724 -0.075632836866
  0.301127490895 -0.152363359938
  0.388472755872 -0.180756951869
  0.249914682911  0.020915538014
  0.059011297430  0.385953039661
  0.003152051704  0.487110565565
  0.000572607872  0.223638693255
 -0.000003253954  0.021457369777
  0.000021068649 -0.001738535917
 -0.000006207536  0.000336961233
   10    1   14
  3.875633130
  1573.111300      295.9291500      108.3458800      47.08844000
  21.82595400      10.48002400      4.988653200      2.304761500
 0.9995225700     0.3810215700
  0.000091140854
  0.002174076220
  0.015331997160
  0.061796932393
  0.166120843734
  0.288032305531
  0.343072997326
  0.296226933977
  0.188748779124
  0.061891705617
*
Spectral Representation Operator
Core primitive basis
Exchange
End of Spectral Representation Operator


/Lu.ECP.Shyichuk.27s23p15d10f.1s1p1d1f.0e-AIMP-Lu2O3-L2.
Orthogonalization AIMP for Lu in C2 site of Ia-3 Lu2O3
Structure: Zeler et al., Materials 7(2014)7059, 10.3390/ma7107059
   3.000    3
    27    1
 0.6701311900E+11  7216717800.      1171529200.      235233120.0
  55877724.00      14949778.00      4363017.000      1375340.500
  456540.5200      160435.3700      59637.10400      23233.14400
  9474.040900      4032.731300      1779.753900      807.8776800
  374.9260700      172.1622700      81.75905400      34.04620200
  17.67359800      7.084092600      3.479507300     0.9931652500
 0.4458321400     0.7721303500E-01 0.3053352200E-01
 -0.000000006602
  0.000000030372
 -0.000000110109
  0.000000467104
 -0.000019814116
 -0.000025438707
 -0.000065020564
 -0.000135458994
 -0.000276173790
 -0.000562074519
 -0.001128751817
 -0.002287964687
 -0.004591280277
 -0.009005425331
 -0.015777495814
```

```
-0.019864620056
 0.003674407076
 0.095669234813
 0.150371765394
-0.208807373642
-0.476513670713
 0.433877955095
 0.774103969652
-0.697134200512
-0.667504003869
 0.018312764687
-0.002250814573
  23    1
 55602773.00      7765745.900      1589584.300      401810.9500
 118603.3400      39300.00000      14430.35400      5793.124600
 2497.821500      1143.717300      551.3977100      277.7501400
 145.0303300      77.44745600      41.75015500      23.22694800
 12.93638700      7.026064900      3.744504700      1.899190800
0.9146347400     0.4226736900     0.1733363600
-0.000000208834
-0.000002193277
-0.000007354534
-0.000016056769
-0.000052296548
-0.000137100123
-0.000384984491
-0.001073007694
-0.002992834083
-0.007820741123
-0.017879727621
-0.031561759905
-0.033702007324
 0.013470382979
 0.109949154142
 0.153801162689
-0.050100480807
-0.270701463686
-0.308966644639
 0.137593116095
 0.511362032010
 0.533283698470
 0.008202581313
  15   1
 16241.49300      4278.988200      1524.597100      640.8577100
 295.5655200      143.5265800      71.89617500      36.65707500
 18.88423700      9.510456800      4.620822600      2.112008600
0.7516843600     0.2514027000     0.7547191500E-01
-0.000041408419
-0.000295443174
-0.001623895508
-0.007256691312
-0.026159266921
-0.075634480574
-0.152365981293
-0.180761366840
 0.020919038527
 0.385960374351
 0.487128098751
 0.223613234532
 0.021440002232
-0.001731526237
 0.000335222591
  10    1
 1573.111300      295.9291500      108.3458800      47.08844000
 21.82595400      10.48002400      4.988653200      2.304761500
0.9995225700     0.3810215700
-0.000091144099
-0.002174256357
-0.015332623865
-0.061801796287
-0.166127393441
```

```
 -0.288057861985
 -0.343067330115
 -0.296269691843
 -0.188555949165
 -0.062090448244
*
* External AIMP:
* Local Pot. Param. :
*                      A(AIMP)=-Zeff*A(ECP)
*
M1
   15
  573250.0000      124682.0000      38556.00000      13233.40000
  4895.600000      1863.700000      729.9500000      332.2300000
  125.6600000      59.61100000      29.10400000      10.59000000
  5.020600000      1.796200000     0.8441200000
 0.2012900221     0.1781175038     0.2895424193     0.4474200300
 0.6139239039     0.7686169723      1.306066160      1.768520444
  1.289621356      4.236266210     0.5430171971      5.140778014
  2.701068441      2.254929883     0.9274881105
M2
    0
COREREP
   1.000
PROJOP
    3
   27    5    2    2    2    2    2
  4672.119192      807.7374734      189.2668345      41.83658654
  7.894165093
 0.6701311900E+11  7216717800.      1171529200.      235233120.0
  55877724.00      14949778.00      4363017.000      1375340.500
  456540.5200      160435.3700      59637.10400      23233.14400
  9474.040900      4032.731300      1779.753900      807.8776800
  374.9260700      172.1622700      81.75905400      34.04620200
  17.67359800      7.084092600      3.479507300     0.9931652500
 0.4458321400     0.7721303500E-01 0.3053352200E-01
 -0.000000244159 -0.000000081509 -0.000000051623  0.000000015328 -0.000000006602
  0.000001120858  0.000000374559  0.000000236647 -0.000000070588  0.000000030372
 -0.000004011432 -0.000001348943 -0.000000839506  0.000000257507 -0.000000110109
  0.000016057858  0.000005559796  0.000003220047 -0.000001123113  0.000000467104
 -0.000595541249 -0.000221485139 -0.000106087817  0.000050449357 -0.000019814116
 -0.000742824066 -0.000280859241 -0.000128808397  0.000065473413 -0.000025438707
 -0.001956848980 -0.000727632203 -0.000351836480  0.000165071405 -0.000065020564
 -0.003949491619 -0.001497904728 -0.000690998559  0.000348559686 -0.000135458994
 -0.008181500871 -0.003073808862 -0.001481836604  0.000700415905 -0.000276173790
 -0.016327088779 -0.006257084040 -0.002933261137  0.001449265364 -0.000562074519
 -0.032284657506 -0.012495618828 -0.005959476262  0.002885500803 -0.001128751817
 -0.063239742391 -0.025221332183 -0.011988747789  0.005864870294 -0.002287964687
 -0.120490513298 -0.050090557969 -0.024128819118  0.011760110244 -0.004591280277
 -0.213402970404 -0.096618047498 -0.046861593352  0.023034061154 -0.009005425331
 -0.315171211007 -0.163495731536 -0.082167305295  0.040358950559 -0.015777495814
 -0.302931771877 -0.195815366279 -0.100636648259  0.050567288681 -0.019864620056
 -0.121186270196  0.010629479687  0.012943773324 -0.008841422187  0.003674407076
 -0.007602139549  0.503630569359  0.431201117837 -0.238269725208  0.095669234813
 -0.000655830866  0.534062027039  0.582640998638 -0.363878354105  0.150371765394
  0.000524008096  0.095859148762 -0.464931045046  0.457452434327 -0.208807373642
 -0.000298564760 -0.014771792765 -0.818865751169  0.986637816361 -0.476513670713
  0.000144200698  0.005047193470 -0.115528069255 -0.614309913294  0.433877955095
 -0.000076444791 -0.002355683479  0.014445973844 -0.857274783949  0.774103969652
  0.000025311037  0.000734658076 -0.003840162497 -0.057272117626 -0.697134200512
 -0.000011469950 -0.000343799497  0.001777508070  0.012687282733 -0.667504003869
  0.000002933541  0.000086221644 -0.000414091756 -0.002175516904  0.018312764687
 -0.000001429602 -0.000040355291  0.000197846194  0.001109383517 -0.002250814573
   23    4    6    6    6    6
  713.1334242      160.1518251      32.05275459      5.345238651
  55602773.00      7765745.900      1589584.300      401810.9500
  118603.3400      39300.00000      14430.35400      5793.124600
  2497.821500      1143.717300      551.3977100      277.7501400
  145.0303300      77.44745600      41.75015500      23.22694800
  12.93638700      7.026064900      3.744504700      1.899190800
 0.9146347400     0.4226736900     0.1733363600
```

```
  0.000001787485 -0.000000941647  0.000000369679 -0.000000208834
  0.000029778262 -0.000014803268  0.000007475706 -0.000002193277
  0.000077473636 -0.000039657056  0.000017831083 -0.000007354534
  0.000219006569 -0.000109129025  0.000055187137 -0.000016056769
  0.000590313057 -0.000299795487  0.000141848105 -0.000052296548
  0.001617915761 -0.000820945041  0.000396173407 -0.000137100123
  0.004448869506 -0.002272298148  0.001092142088 -0.000384984491
  0.012330210848 -0.006350246569  0.003064294793 -0.001073007694
  0.033632733740 -0.017577783195  0.008499455792 -0.002992834083
  0.085361492813 -0.045767069166  0.022290287698 -0.007820741123
  0.184634471289 -0.103019720164  0.050574697895 -0.017879727621
  0.306178024147 -0.179921934887  0.089738796178 -0.031561759905
  0.333940331973 -0.192209881343  0.094506777844 -0.033702007324
  0.191850811537  0.015577864157 -0.030481842688  0.013470382979
  0.041370607946  0.389528167148 -0.292149788646  0.109949154142
  0.001846179756  0.478691458477 -0.385613764677  0.153801162689
  0.000145645400  0.202680814722  0.045163305907 -0.050100480807
 -0.000133674685  0.026110541880  0.546227868295 -0.270701463686
 -0.000015763674  0.001456506886  0.481895911822 -0.308966644639
 -0.000005991985  0.000167738118  0.118341386570  0.137593116095
  0.000001325054 -0.000006645139  0.003751839376  0.511362032010
  0.000000049509  0.000007899000  0.001517632444  0.533283698470
 -0.000000140365 -0.000007999343 -0.000612278648  0.008202581313
   15    2   10   10
  123.9726510      18.81588797
  16241.49300      4278.988200      1524.597100      640.8577100
  295.5655200      143.5265800      71.89617500      36.65707500
  18.88423700      9.510456800      4.620822600      2.112008600
 0.7516843600     0.2514027000     0.7547191500E-01
  0.000085227100 -0.000041408419
  0.000604044474 -0.000295443174
  0.003323658643 -0.001623895508
  0.014681012364 -0.007256691312
  0.052573221249 -0.026159266921
  0.148494384703 -0.075634480574
  0.301127538751 -0.152365981293
  0.388472903140 -0.180761366840
  0.249914734257  0.020919038527
  0.059010929176  0.385960374351
  0.003151889465  0.487128098751
  0.000572512361  0.223613234532
 -0.000003200949  0.021440002232
  0.000021057129 -0.001731526237
 -0.000006203861  0.000335222591
   10    1   14
  4.158760063
  1573.111300      295.9291500      108.3458800      47.08844000
  21.82595400      10.48002400      4.988653200      2.304761500
 0.9995225700     0.3810215700
 -0.000091144099
 -0.002174256357
 -0.015332623865
 -0.061801796287
 -0.166127393441
 -0.288057861985
 -0.343067330115
 -0.296269691843
 -0.188555949165
 -0.062090448244
*
Spectral Representation Operator
Core primitive basis
Exchange
End of Spectral Representation Operator

/Lu.ECP.Shyichuk.0s.0s.0e-AIMP-Lu2O3-L1.
AIMP for Lu in C3i site of Ia-3 Lu2O3
Structure: Zeler et al., Materials 7(2014)7059, 10.3390/ma7107059
   3.000    0
    0    0
*
```

```
* External AIMP:
* Local Pot. Param. :
*                    A(AIMP)=-Zeff*A(ECP)
*
M1
   15
  574860.0000      125310.0000      38821.00000      13344.00000
  4942.100000      1882.900000      735.9700000      333.9200000
  126.0400000      59.54900000      28.74600000      10.60800000
  5.086000000      1.844700000     0.8846600000
 0.2009213796     0.1773913013     0.2881511987     0.4452263163
 0.6117365920     0.7662284548      1.300997964      1.777353987
  1.294956501      4.250875541     0.5311111846      5.114314469
  2.682519666      2.219849107      1.005033004
M2
    0
COREREP
   1.000
PROJOP
    3
   27    5    2    2    2    2    2
  4671.835144      807.4535105      188.9828466      41.55288421
  7.612401544
 0.6701311900E+11  7216717800.      1171529200.      235233120.0
  55877724.00      14949778.00      4363017.000      1375340.500
  456540.5200      160435.3700      59637.10400      23233.14400
  9474.040900      4032.731300      1779.753900      807.8776800
  374.9260700      172.1622700      81.75905400      34.04620200
  17.67359800      7.084092600      3.479507300     0.9931652500
 0.4458321400     0.7721303500E-01 0.3053352200E-01
 -0.000000244159 -0.000000081509 -0.000000051623  0.000000015328 -0.000000006602
  0.000001120858  0.000000374559  0.000000236647 -0.000000070588  0.000000030374
 -0.000004011432 -0.000001348943 -0.000000839506  0.000000257506 -0.000000110116
  0.000016057858  0.000005559797  0.000003220047 -0.000001123109  0.000000467131
 -0.000595541247 -0.000221485142 -0.000106087807  0.000050449202 -0.000019814395
 -0.000742824063 -0.000280859243 -0.000128808383  0.000065473198 -0.000025438669
 -0.001956848975 -0.000727632218 -0.000351836447  0.000165070969 -0.000065022341
 -0.003949491597 -0.001497904726 -0.000690998486  0.000348558245 -0.000135455942
 -0.008181500877 -0.003073808998 -0.001481836453  0.000700415445 -0.000276195204
 -0.016327088540 -0.006257083642 -0.002933260892  0.001449252122 -0.000561989435
 -0.032284657317 -0.012495618809 -0.005959475717  0.002885488408 -0.001128728096
 -0.063239741794 -0.025221331465 -0.011988746597  0.005864833971 -0.002287802822
 -0.120490512185 -0.050090556902 -0.024128817125  0.011760042489 -0.004591001203
 -0.213402967907 -0.096618044489 -0.046861587674  0.023033930823 -0.009004931174
 -0.315171204704 -0.163495724269 -0.082167298278  0.040358698261 -0.015776321530
 -0.302931771382 -0.195815360358 -0.100636631151  0.050567030453 -0.019863918091
 -0.121186283481  0.010629428500  0.012943715203 -0.008841450911  0.003675558178
 -0.007602141998  0.503630591588  0.431201189209 -0.238268061028  0.095659912538
 -0.000655828604  0.534062026008  0.582640824847 -0.363876452546  0.150368486923
  0.000524010169  0.095859164291 -0.464931114345  0.457450390598 -0.208811271663
 -0.000298565536 -0.014771785989 -0.818865068909  0.986626606953 -0.476439144083
  0.000144199912  0.005047171875 -0.115528665973 -0.614292526307  0.433749447001
 -0.000076444751 -0.002355681607  0.014445926463 -0.857273926704  0.774126811080
  0.000025311211  0.000734669729 -0.003840156887 -0.057286720821 -0.696902139902
 -0.000011469543 -0.000343809841  0.001777728159  0.012691608588 -0.667784548498
  0.000002932694  0.000086247918 -0.000414561761 -0.002181867063  0.018649282941
 -0.000001429421 -0.000040360587  0.000197945719  0.001110176627 -0.002574922135
   23    4    6    6    6    6
  712.8494695      159.8678645      31.76916200      5.063535877
  55602773.00      7765745.900      1589584.300      401810.9500
  118603.3400      39300.00000      14430.35400      5793.124600
  2497.821500      1143.717300      551.3977100      277.7501400
  145.0303300      77.44745600      41.75015500      23.22694800
  12.93638700      7.026064900      3.744504700      1.899190800
 0.9146347400     0.4226736900     0.1733363600
  0.000001787485 -0.000000941647 -0.000000369676 -0.000000211731
  0.000029778262 -0.000014803267 -0.000007475672 -0.000002223711
  0.000077473637 -0.000039657055 -0.000017830965 -0.000007456779
  0.000219006573 -0.000109129009 -0.000055186956 -0.000016278231
  0.000590313060 -0.000299795562 -0.000141846684 -0.000053035340
  0.001617915777 -0.000820945151 -0.000396170308 -0.000139018757
```

```
  0.004448869540 -0.002272298527 -0.001092132632 -0.000390410663
  0.012330210985 -0.006350247575 -0.003064269999 -0.001088011353
  0.033632733937 -0.017577786075 -0.008499381949 -0.003035086569
  0.085361492404 -0.045767075842 -0.022290108595 -0.007929887039
  0.184634467298 -0.103019734929 -0.050574247850 -0.018132873849
  0.306178043485 -0.179921982031 -0.089738115133 -0.031999439549
  0.333940359115 -0.192209940929 -0.094505831349 -0.034189488144
  0.191850774038  0.015578030291  0.030481272923  0.013693235271
  0.041370581168  0.389528330318  0.292147926177  0.111419748918
  0.001846184747  0.478691276674  0.385608279752  0.156109722792
  0.000145653423  0.202680590145 -0.045159697777 -0.051078449881
 -0.000133664932  0.026110805231 -0.546223724282 -0.274078472763
 -0.000015766198  0.001456433841 -0.481882985622 -0.313953683951
 -0.000005991598  0.000167969926 -0.118359068355  0.140504495696
  0.000001323965 -0.000007282852 -0.003754261733  0.517074336971
  0.000000080299  0.000008479797 -0.001524517978  0.543180129564
 -0.000000146269 -0.000008211187  0.000618555432 -0.015735095708
   15    2   10   10
  123.6886766      18.53239027
  16241.49300      4278.988200      1524.597100      640.8577100
  295.5655200      143.5265800      71.89617500      36.65707500
  18.88423700      9.510456800      4.620822600      2.112008600
 0.7516843600     0.2514027000     0.7547191500E-01
  0.000085227076 -0.000041407692
  0.000604044322 -0.000295436661
  0.003323657658 -0.001623867413
  0.014681007630 -0.007256532095
  0.052573202795 -0.026158801412
  0.148494365724 -0.075632836866
  0.301127490895 -0.152363359938
  0.388472755872 -0.180756951869
  0.249914682911  0.020915538014
  0.059011297430  0.385953039661
  0.003152051704  0.487110565565
  0.000572607872  0.223638693255
 -0.000003253954  0.021457369777
  0.000021068649 -0.001738535917
 -0.000006207536  0.000336961233
   10    1   14
  3.875633130
  1573.111300      295.9291500      108.3458800      47.08844000
  21.82595400      10.48002400      4.988653200      2.304761500
 0.9995225700     0.3810215700
  0.000091140854
  0.002174076220
  0.015331997160
  0.061796932393
  0.166120843734
  0.288032305531
  0.343072997326
  0.296226933977
  0.188748779124
  0.061891705617
*
Spectral Representation Operator
Core primitive basis
Exchange
End of Spectral Representation Operator


/Lu.ECP.Shyichuk.0s.0s.0e-AIMP-Lu2O3-L2.
AIMP for Lu in C2 site of Ia-3 Lu2O3
Structure: Zeler et al., Materials 7(2014)7059, 10.3390/ma7107059
   3.000    0
    0    0
*
* External AIMP:
* Local Pot. Param. :
*                      A(AIMP)=-Zeff*A(ECP)
*
M1
```

```
   15
  573250.0000      124682.0000      38556.00000      13233.40000
  4895.600000      1863.700000      729.9500000      332.2300000
  125.6600000      59.61100000      29.10400000      10.59000000
  5.020600000      1.796200000     0.8441200000
 0.2012900221     0.1781175038     0.2895424193     0.4474200300
 0.6139239039     0.7686169723      1.306066160      1.768520444
  1.289621356      4.236266210     0.5430171971      5.140778014
  2.701068441      2.254929883     0.9274881105
M2
    0
COREREP
   1.000
PROJOP
    3
   27    5    2    2    2    2    2
  4672.119192      807.7374734      189.2668345      41.83658654
  7.894165093
 0.6701311900E+11  7216717800.      1171529200.      235233120.0
  55877724.00      14949778.00      4363017.000      1375340.500
  456540.5200      160435.3700      59637.10400      23233.14400
  9474.040900      4032.731300      1779.753900      807.8776800
  374.9260700      172.1622700      81.75905400      34.04620200
  17.67359800      7.084092600      3.479507300     0.9931652500
 0.4458321400     0.7721303500E-01 0.3053352200E-01
 -0.000000244159 -0.000000081509 -0.000000051623  0.000000015328 -0.000000006602
  0.000001120858  0.000000374559  0.000000236647 -0.000000070588  0.000000030372
 -0.000004011432 -0.000001348943 -0.000000839506  0.000000257507 -0.000000110109
  0.000016057858  0.000005559796  0.000003220047 -0.000001123113  0.000000467104
 -0.000595541249 -0.000221485139 -0.000106087817  0.000050449357 -0.000019814116
 -0.000742824066 -0.000280859241 -0.000128808397  0.000065473413 -0.000025438707
 -0.001956848980 -0.000727632203 -0.000351836480  0.000165071405 -0.000065020564
 -0.003949491619 -0.001497904728 -0.000690998559  0.000348559686 -0.000135458994
 -0.008181500871 -0.003073808862 -0.001481836604  0.000700415905 -0.000276173790
 -0.016327088779 -0.006257084040 -0.002933261137  0.001449265364 -0.000562074519
 -0.032284657506 -0.012495618828 -0.005959476262  0.002885500803 -0.001128751817
 -0.063239742391 -0.025221332183 -0.011988747789  0.005864870294 -0.002287964687
 -0.120490513298 -0.050090557969 -0.024128819118  0.011760110244 -0.004591280277
 -0.213402970404 -0.096618047498 -0.046861593352  0.023034061154 -0.009005425331
 -0.315171211007 -0.163495731536 -0.082167305295  0.040358950559 -0.015777495814
 -0.302931771877 -0.195815366279 -0.100636648259  0.050567288681 -0.019864620056
 -0.121186270196  0.010629479687  0.012943773324 -0.008841422187  0.003674407076
 -0.007602139549  0.503630569359  0.431201117837 -0.238269725208  0.095669234813
 -0.000655830866  0.534062027039  0.582640998638 -0.363878354105  0.150371765394
  0.000524008096  0.095859148762 -0.464931045046  0.457452434327 -0.208807373642
 -0.000298564760 -0.014771792765 -0.818865751169  0.986637816361 -0.476513670713
  0.000144200698  0.005047193470 -0.115528069255 -0.614309913294  0.433877955095
 -0.000076444791 -0.002355683479  0.014445973844 -0.857274783949  0.774103969652
  0.000025311037  0.000734658076 -0.003840162497 -0.057272117626 -0.697134200512
 -0.000011469950 -0.000343799497  0.001777508070  0.012687282733 -0.667504003869
  0.000002933541  0.000086221644 -0.000414091756 -0.002175516904  0.018312764687
 -0.000001429602 -0.000040355291  0.000197846194  0.001109383517 -0.002250814573
   23    4    6    6    6    6
  713.1334242      160.1518251      32.05275459      5.345238651
  55602773.00      7765745.900      1589584.300      401810.9500
  118603.3400      39300.00000      14430.35400      5793.124600
  2497.821500      1143.717300      551.3977100      277.7501400
  145.0303300      77.44745600      41.75015500      23.22694800
  12.93638700      7.026064900      3.744504700      1.899190800
 0.9146347400     0.4226736900     0.1733363600
  0.000001787485 -0.000000941647  0.000000369679 -0.000000208834
  0.000029778262 -0.000014803268  0.000007475706 -0.000002193277
  0.000077473636 -0.000039657056  0.000017831083 -0.000007354534
  0.000219006569 -0.000109129025  0.000055187137 -0.000016056769
  0.000590313057 -0.000299795487  0.000141848105 -0.000052296548
  0.001617915761 -0.000820945041  0.000396173407 -0.000137100123
  0.004448869506 -0.002272298148  0.001092142088 -0.000384984491
  0.012330210848 -0.006350246569  0.003064294793 -0.001073007694
  0.033632733740 -0.017577783195  0.008499455792 -0.002992834083
  0.085361492813 -0.045767069166  0.022290287698 -0.007820741123
  0.184634471289 -0.103019720164  0.050574697895 -0.017879727621
```

```
  0.306178024147 -0.179921934887  0.089738796178 -0.031561759905
  0.333940331973 -0.192209881343  0.094506777844 -0.033702007324
  0.191850811537  0.015577864157 -0.030481842688  0.013470382979
  0.041370607946  0.389528167148 -0.292149788646  0.109949154142
  0.001846179756  0.478691458477 -0.385613764677  0.153801162689
  0.000145645400  0.202680814722  0.045163305907 -0.050100480807
 -0.000133674685  0.026110541880  0.546227868295 -0.270701463686
 -0.000015763674  0.001456506886  0.481895911822 -0.308966644639
 -0.000005991985  0.000167738118  0.118341386570  0.137593116095
  0.000001325054 -0.000006645139  0.003751839376  0.511362032010
  0.000000049509  0.000007899000  0.001517632444  0.533283698470
 -0.000000140365 -0.000007999343 -0.000612278648  0.008202581313
   15    2   10   10
  123.9726510      18.81588797
  16241.49300      4278.988200      1524.597100      640.8577100
  295.5655200      143.5265800      71.89617500      36.65707500
  18.88423700      9.510456800      4.620822600      2.112008600
 0.7516843600     0.2514027000     0.7547191500E-01
  0.000085227100 -0.000041408419
  0.000604044474 -0.000295443174
  0.003323658643 -0.001623895508
  0.014681012364 -0.007256691312
  0.052573221249 -0.026159266921
  0.148494384703 -0.075634480574
  0.301127538751 -0.152365981293
  0.388472903140 -0.180761366840
  0.249914734257  0.020919038527
  0.059010929176  0.385960374351
  0.003151889465  0.487128098751
  0.000572512361  0.223613234532
 -0.000003200949  0.021440002232
  0.000021057129 -0.001731526237
 -0.000006203861  0.000335222591
   10    1   14
  4.158760063
  1573.111300      295.9291500      108.3458800      47.08844000
  21.82595400      10.48002400      4.988653200      2.304761500
 0.9995225700     0.3810215700
 -0.000091144099
 -0.002174256357
 -0.015332623865
 -0.061801796287
 -0.166127393441
 -0.288057861985
 -0.343067330115
 -0.296269691843
 -0.188555949165
 -0.062090448244
*
Spectral Representation Operator
Core primitive basis
Exchange
End of Spectral Representation Operator



/O.ECP.Shyichuk.0s.0s.0e-AIMP-Lu2O3-O.
AIMP for O in Ia-3 Lu2O3
Structure: Zeler et al., Materials 7(2014)7059, 10.3390/ma7107059
  -2.000    0
    0    0
*
* External AIMP:
* Local Pot. Param. :
*                     A(AIMP)=-Zeff*A(ECP)
*
M1
   15
  397010.0000      63067.00000      14702.00000      3955.200000
  1221.900000      421.8700000      157.9000000      63.50900000
  26.55600000      10.24100000      4.012500000      1.833900000
```

```
 0.7704600000     0.2324500000     0.2323900000
 -.1755357730E-01 -.2071123151E-01 -.3845382872E-01 -.6727596366E-01
 -.1102820422     -.1801437101     -.2862630463     -.3976737177
 -.4125278236     -.3848289170     -.7072337737     -.9696056635
 -1.413872713      12.90619523     -12.89976923
M2
    0
COREREP
   1.000
PROJOP
    1
   12    2    2    2
  40.66398635      2.403182426
  109234.9000      15421.33000      3272.828000      870.1072000
  268.2706000      91.49282000      33.61740000      13.04648000
  5.203523000      1.477429000     0.5777521000     0.2126743000
  0.000121613287  0.000025884060
  0.000539616262  0.000117867615
  0.002449955450  0.000509519088
  0.010259822357  0.002308467635
  0.037781449934  0.007830228630
  0.116522068974  0.028318105972
  0.277252456278  0.063208928769
  0.421126356425  0.154489280396
  0.268163760351  0.095744871573
  0.024823325246 -0.111360500001
 -0.003681020897 -0.730527964423
  0.001348692500 -0.260157973252
    8    1    6
 0.9623411839
  366.4067000      66.27956000      18.74558000      6.426860000
  2.444513000     0.9577170000     0.3688401000     0.1364496000
  0.000369859963
  0.003795680307
  0.024897031024
  0.073665043290
  0.236201896353
  0.205872842452
  0.706468634543
 -0.071524192944
*
Spectral Representation Operator
Core primitive basis
Exchange
End of Spectral Representation Operator
*
* ==============================================================
* ======               End of AIMPLIB file               ======
* ==============================================================
```

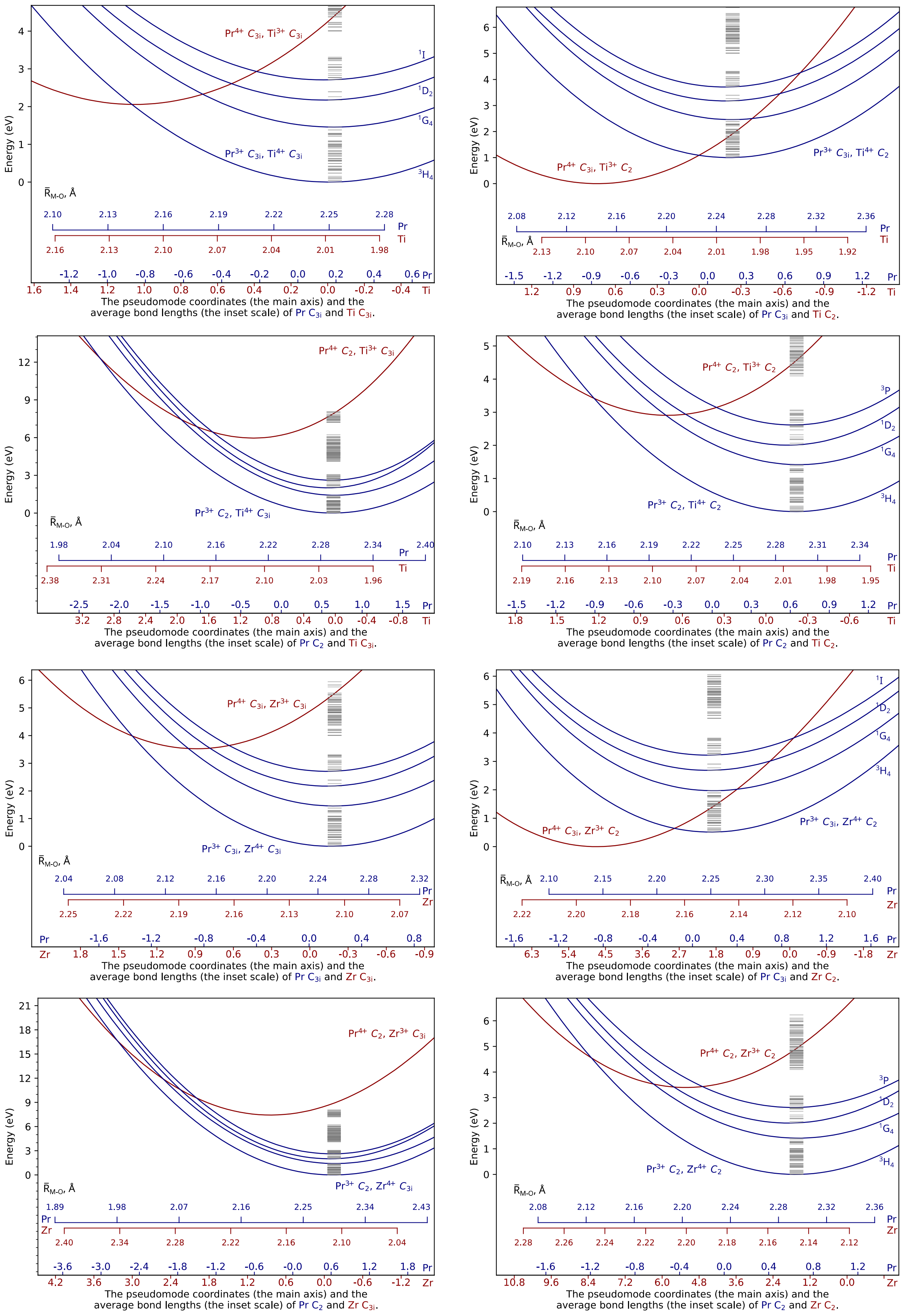


Figure S1. Configurational diagrams for the Pr-Ti and Pr-Zr pairs.

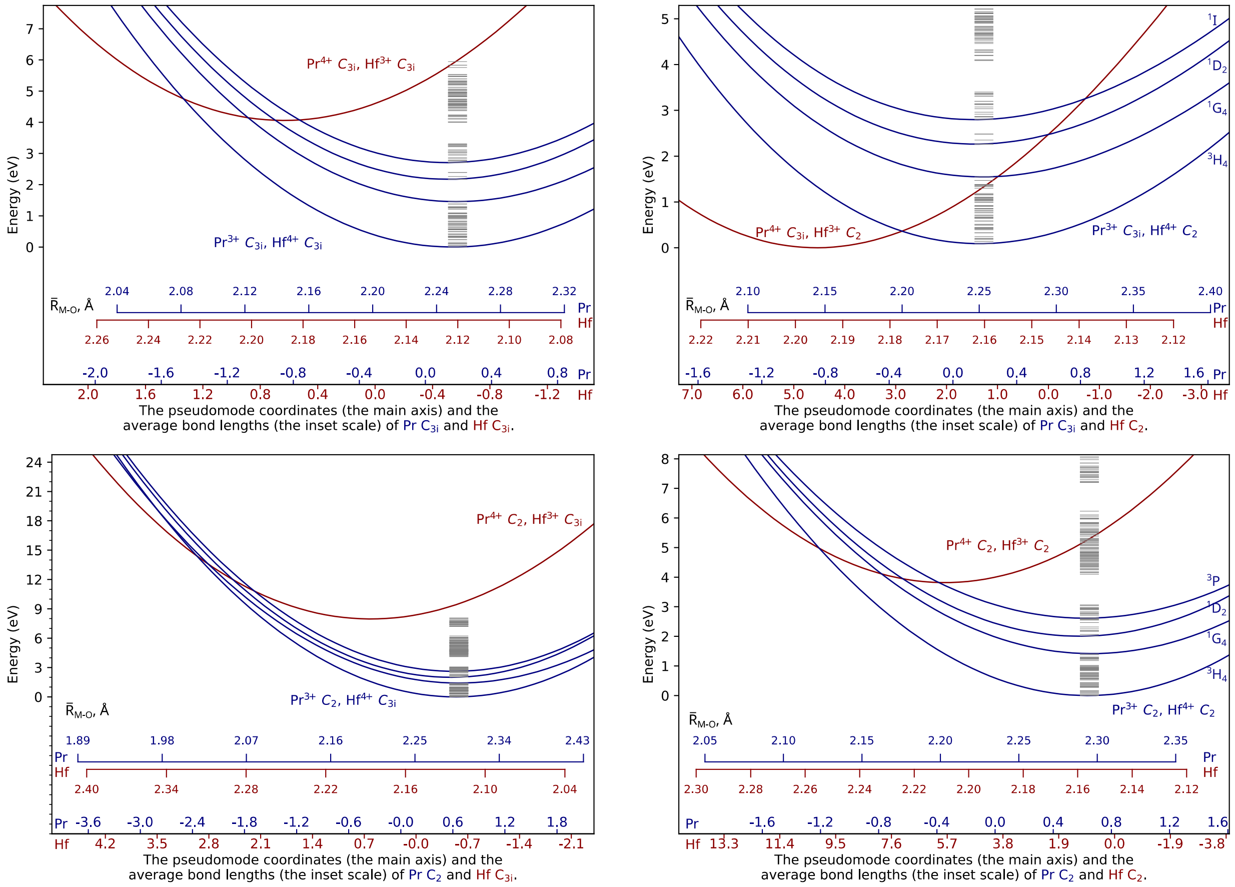


Figure S2. Configurational diagrams for the Pr-Hf pairs ($Hf^{4+}$ PP).

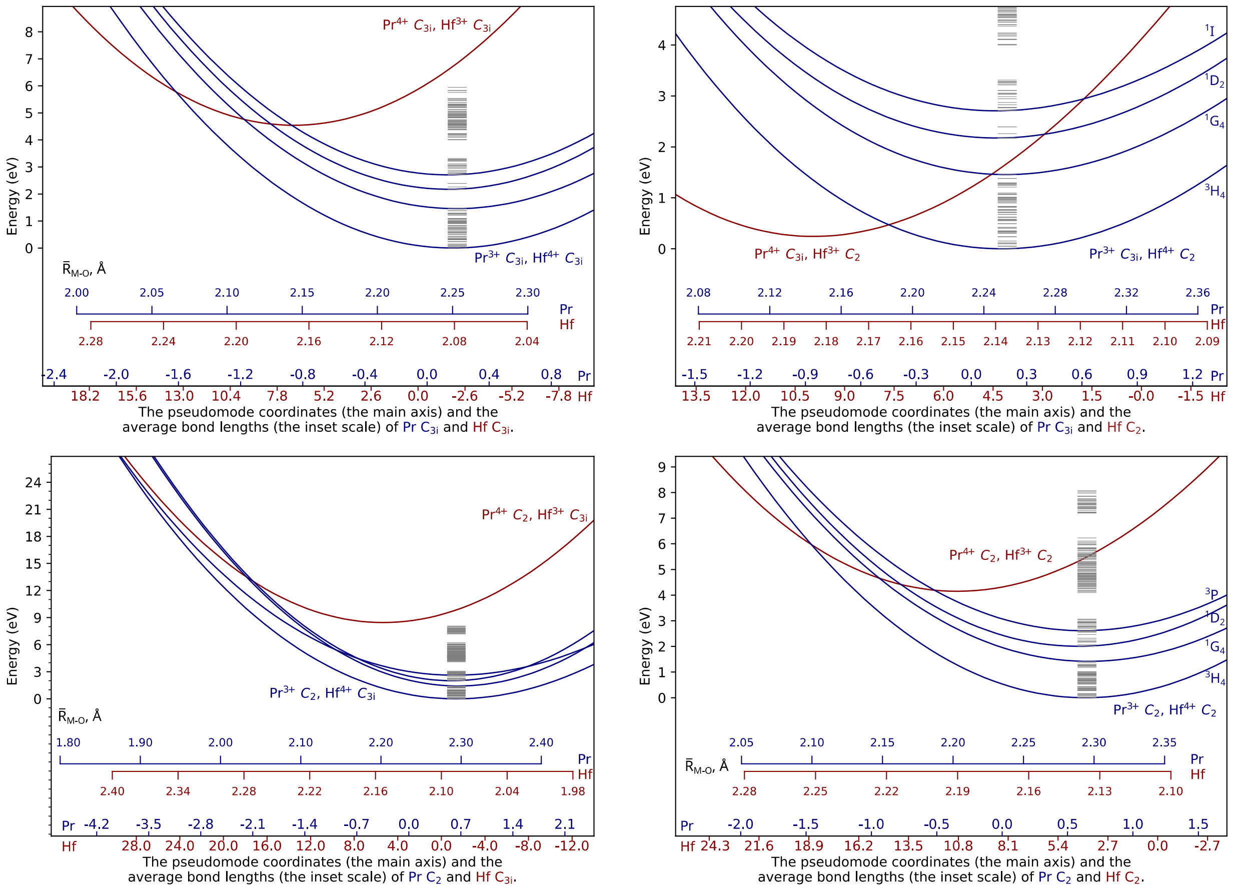


Figure S3. Configurational diagrams for the Pr-Hf pairs ($Hf^{0}$ PP).
